# Gel-Based Triboelectric Nanogenerators for Flexible Sensing: Principles, Properties, and Applications

**DOI:** 10.1007/s40820-024-01432-2

**Published:** 2024-05-31

**Authors:** Peng Lu, Xiaofang Liao, Xiaoyao Guo, Chenchen Cai, Yanhua Liu, Mingchao Chi, Guoli Du, Zhiting Wei, Xiangjiang Meng, Shuangxi Nie

**Affiliations:** 1 School of Light Industry and Food Engineering, Guangxi University, Nanning, 530004 People’s Republic of China; 2 Beijing Institute of Nanoenergy and Nanosystems, Chinese Academy of Sciences, Beijing, 101400 People’s Republic of China

**Keywords:** Triboelectric nanogenerators, Gel materials, Triboelectric materials, Flexible sensing

## Abstract

The rapid development of the Internet of Things and artificial intelligence technologies has increased the need for wearable, portable, and self-powered flexible sensing devices. Triboelectric nanogenerators (TENGs) based on gel materials (with excellent conductivity, mechanical tunability, environmental adaptability, and biocompatibility) are considered an advanced approach for developing a new generation of flexible sensors. This review comprehensively summarizes the recent advances in gel-based TENGs for flexible sensors, covering their principles, properties, and applications. Based on the development requirements for flexible sensors, the working mechanism of gel-based TENGs and the characteristic advantages of gels are introduced. Design strategies for the performance optimization of hydrogel-, organogel-, and aerogel-based TENGs are systematically summarized. In addition, the applications of gel-based TENGs in human motion sensing, tactile sensing, health monitoring, environmental monitoring, human–machine interaction, and other related fields are summarized. Finally, the challenges of gel-based TENGs for flexible sensing are discussed, and feasible strategies are proposed to guide future research.

## Highlights

- Typical structures/working mechanisms of gel-based triboelectric nanogenerators and performance advantages of gel materials reviewed.
- Optimization of hydrogels, organogels, and aerogels for triboelectric nanogenerators in flexible sensing summarized.
- Applications, challenges, and future development directions of gel-based triboelectric nanogenerators in flexible sensing are discussed.

## Graphical abstract

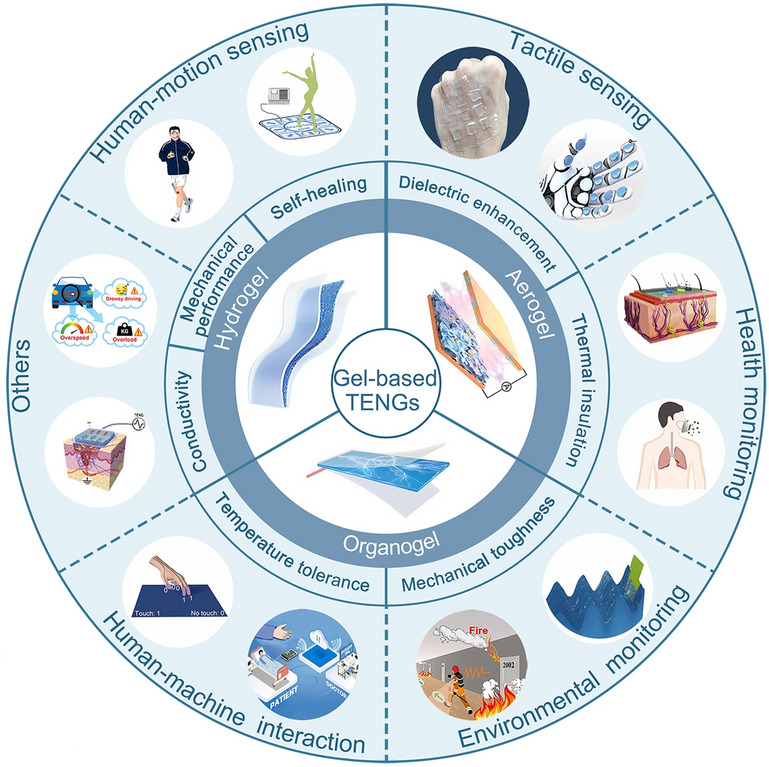

## Introduction

Extensive interest in the Internet of Things and artificial intelligence has prompted the rapid development of flexible sensing technology [1–4], such as wearable electronics, electronic skin, and implantable medical devices [5–10]. To promote practical applications, there is an urgent need for flexible sensors that are wearable, portable, and self-powered. However, the development requirements of the new generation of flexible electronic devices are not met by traditional piezoresistive or capacitive sensors, which require an external power supply, or piezoelectric sensors, which have a relatively restricted selection of materials [11–14]. Triboelectric nanogenerators (TENGs), which based on both contact electrification and electrostatic induction, offer significant advantages for the development of new-generation sensors [15–26]. TENGs are considered an ideal choice for flexible sensors because of their many advantages, e.g., self-powering, compact size, low cost, wide variety of suitable materials, and high sensitivity [27–37]. Materials for flexible sensors must meet a range of strict requirements, such as flexibility, biocompatibility, and environmental tolerance. Therefore, flexible materials with tunable performance are being developed to meet the needs of TENGs for flexible sensing applications.

Gels are three-dimensional network structures composed of particles or polymers within a certain size range dispersed in another medium [38]. Depending on the nature of the solute (water, organic liquids, and gases), gels are categorized as hydrogels, organogels, or aerogels, respectively [39]. The three-dimensional dynamic network structure enables the development of flexible and tailorable gel materials. Gel materials are characterized by excellent conductivity, mechanical flexibility, self-healing ability, environmental adaptability, and biocompatibility [40–42]. Owing to these excellent properties, gel materials have been widely utilized in many fields, including environmental management [43–47], flexible sensing [48–50], electrical engineering [51–53], actuators [54–56], and biomedicine [57–64]. In the field of TENGs, these three types of gels have unique properties and advantages that can be modified for the development and application of various flexible triboelectric sensors.

**Fig. 1:**
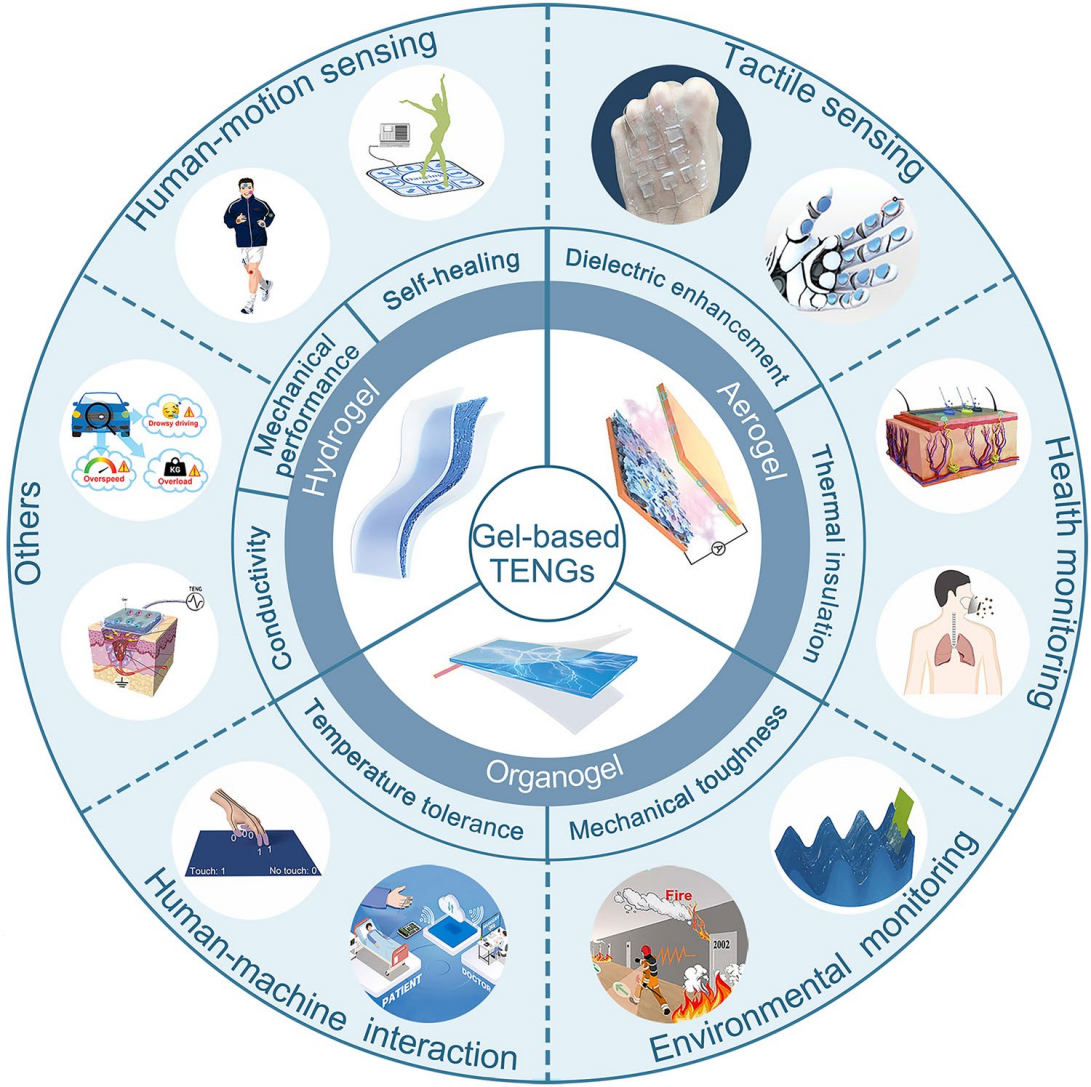
Gel-based TENGs for flexible sensing. Reproduced with permission from Ref. [87], Copyright 2022, Elsevier; Ref. [88], Copyright 2023, Elsevier; Ref. [89], Copyright 2017, American Association for the Advancement of Science; Ref. [90], Copyright 2023, Wiley–VCH.; Ref. [91], Copyright 2022, Wiley–VCH.; Ref. [92], Copyright 2022, Elsevier; Ref. [93], Copyright 2022, Elsevier; Ref. [94], Copyright 2023, Elsevier; Ref [95]. Copyright 2023, Elsevier; Ref. [96], Copyright 2021, Elsevier; Ref. [97], Copyright 2021, Elsevier; Ref. [98], Copyright 2022, American Chemical Society; Ref. [99], Copyright 2023, Wiley–VCH.; Ref. [86], Copyright 2023, Wiley–VCH.; Ref. [100], Copyright 2021, Elsevier

Since Xu et al. first used hydrogel materials in TENGs [65], gel-based TENGs have been extensively studied. Accordingly, summarizing relevant studies on gel-based TENGs highlighted the remarkable results that have been achieved. However, most of these comprehensive reports focused on a single type of gel, predominantly hydrogels [66–71]. Notably, in recent years, significant research has been conducted on organogel- and aerogel-based TENGs to improve their output, environmental adaptability, and mechanical durability [31, 70, 72–86]. These advancements have demonstrated their indispensable position and advantages in the field of flexible sensing. Therefore, it is necessary to review the remarkable advancements in all gel-based TENGs for flexible sensing. This review comprehensively summarizes the research progress in gel-based TENGs for flexible sensing from the perspective of principles, properties, and applications (Fig. 1). First, the basic principles of gel-based TENGs and characteristic advantages of gel materials are briefly introduced. Subsequently, the design strategies for optimizing the performance of hydrogel-, organogel-, and aerogel-based TENGs are systematically summarized. Subsequently, the applications of gel-based TENGs in human motion sensing, tactile sensing, health monitoring, environmental monitoring, human–machine interactions, and other fields are outlined in detail. Finally, considering the current challenges of gel-based TENGs, viable strategies are proposed, and the prospects of gel-based TENGs in flexible sensing are discussed.

## Working Mechanism and Advantages of Gel-Based TENGs

Gel-based TENGs can function properly under deformation states such as stretching, bending, and folding. Combining gels and TENGs can fully leverage the advantages of both and is an effective strategy for fabricating high-performance and sustainable flexible devices. This section begins with a brief overview of TENGs, followed by a discussion of the performance advantages of gel materials and the typical structure and working mechanism of gel-based TENGs.

### Overview of TENGs

**Fig. 2:**
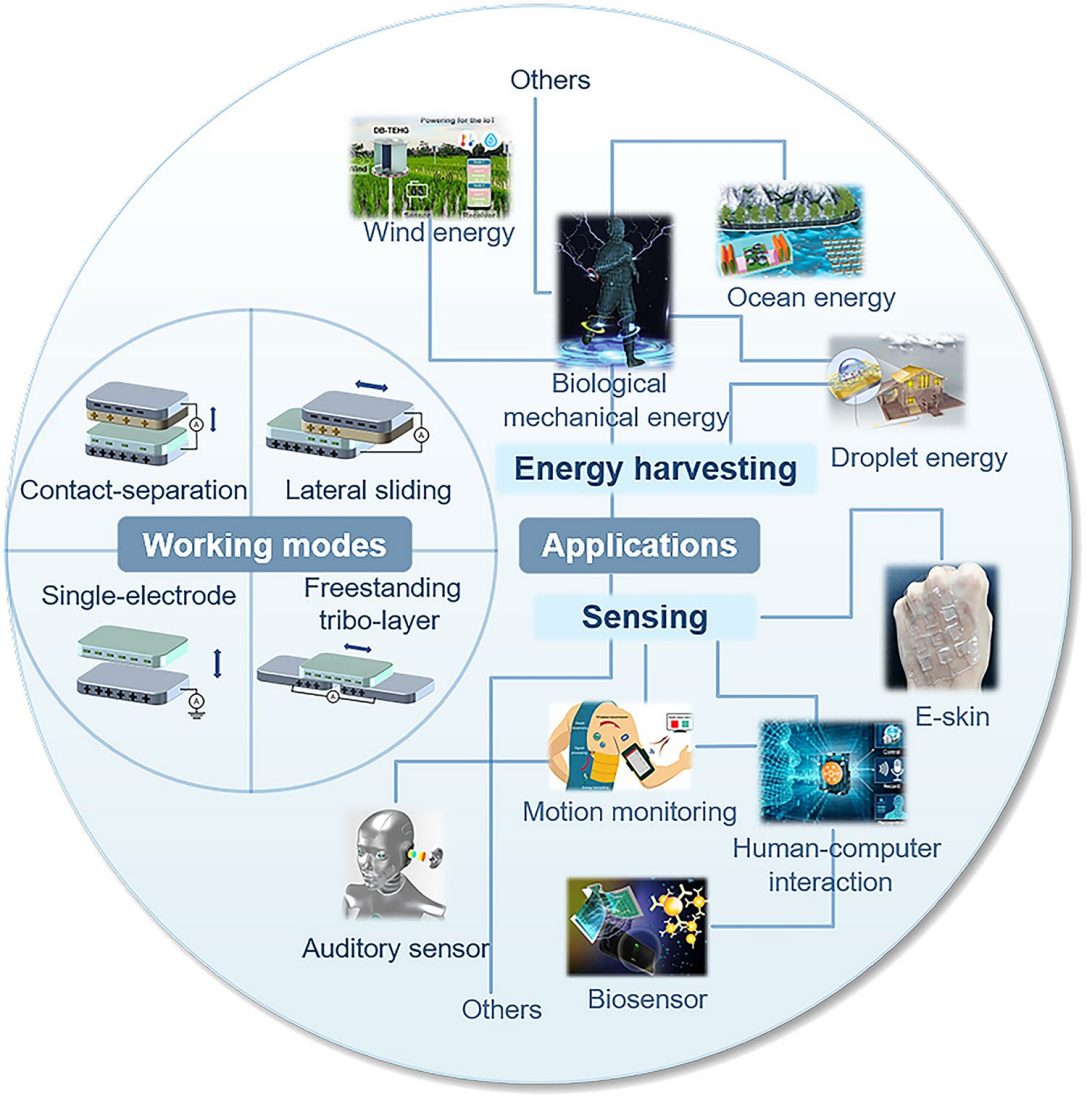
Working modes and applications of TENGs. Reproduced with permission from Ref. [111], Copyright 2022, Elsevier; Ref. [112], Copyright 2023, Elsevier; Ref. [113], Copyright 2023, Wiley–VCH; Ref. [116], Copyright 2023, Wiley–VCH; Ref. [89], Copyright 2017, American Association for the Advancement of Science; Ref. [122], Copyright 2023, American Chemical Society; Ref. [123], Copyright 2020, American Association for the Advancement of Science; Ref. [125], Copyright 2020, Elsevier; Ref. [117], Copyright 2018, American Association for the Advancement of Science

TENGs were first proposed by Wang et al. in 2012 [16], and the related theory was derived from Maxwell’s equations. TENGs are a type of distributed energy-harvesting device based on the coupling effect of contact electrification and electrostatic induction [24]. According to the requirements of practical applications, TENGs are classified into four basic working modes: vertical contact–separation [101, 102], lateral sliding [103], single-electrode [104], and freestanding tribolayer [105]. TENGs are broadly applicable and are classified into two main categories: energy harvesting and sensing. TENGs efficiently use dispersed low-frequency energy sources such as biomechanical, wind, ocean, and water wave energy to achieve energy conversion and harvesting [106–116]. The miniaturization ability and flexibility of TENGs offer unique advantages in sensing applications, such as human motion, biomechanics, and human–computer interfaces [89, 117–131]. Figure 2 illustrates the four working modes and applications of TENGs.

### Advantages of Gel Materials for TENGs

Various flexible materials have been explored for the development of TENGs for flexible sensor applications. Films [132–135], fibers [136], membranes [137], and gels are commonly used as flexible materials. Films or membranes are most widely used in TENGs owing to their good flexibility and mature preparation processes. For example, polydimethylsiloxane (PDMS) films and membranes have played an invaluable role in the development of flexible TENGs. Strategies such as MXene doping, pre-stretching, ultraviolet ozone irradiation [132], or chemical modification treatments [133], can effectively improve the applicability of PDMS in TENGs for flexible sensing. Films and membranes respond better to external forces under localized deformation and smaller stretching conditions. However, in larger mechanical deformation and impact environments, strong stretching tends to cause irreversible damage to the mechanical properties and electrical conductivity of the material, thus limiting the sensing applications of the film- and membrane-based TENGs [138]. Fibers have a high aspect ratio and specific surface area, excellent flexibility and ductility [136]. However, fibers are limited by the intrinsic conductive properties of the material, and complex structural designs (weaving, knitting, serpentine sewing, and spiral winding) are often required to prepare fiber-based TENGs for flexible sensing [136]. In contrast, gel materials with a dynamic three-dimensional network structure have excellent performance tunability, which is especially suitable for flexible TENGs. Gels can achieve the integration of flexibility and conductivity on the gel substrate, on-demand and adaptable designs in terms of transparency, interfacial adhesion, self-healing, and extreme environmental tolerance. For instance, a conductive, transparent, and self-cleaning polyacrylamide (PAM) hydrogel with high mechanical stability was combined with a TENG for wireless communication sensing [139]. In addition, a highly conductive, self-healing, and freeze-resistant gelatin organic hydrogel-based TENG [140], and a highly conductive, ultralight, and thermally insulating calcium alginate aerogel-based TENG were demonstrated for high-temperature environmental sensing [74]. The advantages of gel materials, such as their high conductivity, mechanical toughness, self-healing ability, and excellent environmental adaptability make them suitable for the development of wearable, portable, and self-powered flexible sensors. Flexible triboelectric sensors based on gel materials allow a greater range and complexity of deformation, resulting in more sensitive sensing and response to environmental changes.

The material composition and structure of gel materials can be tailored to meet the requirements of different flexible TENGs sensing applications. Table 1 summarizes the advantages and disadvantages of the three types of gels used in TENGs and sensing applications. Hydrogels can be used as flexible substrates for TENG-based sensors because of their good flexibility and tunable mechanical properties. They are particularly suitable for applications with frequent deformation and stress changes, such as electronic skin and wearable sensors [65]. In addition, conductive gels fabricated using intrinsically conductive materials or conductive enhancers can be used as flexible electrodes in TENGs. TENGs based on conductive gels with the dual functions of flexibility and conductivity can overcome the problem of conductive failure caused by the rupture of traditional electrode materials under stretching and greatly expand the application scope of TENGs in flexible sensing. Currently, hydrogels exhibit the greatest advantages for application in TENG electrodes. Ultra-strong and ultra-tough conductive gels can be prepared by combining traditional gel materials (e.g., polyvinyl alcohol (PVA), and PAM) prepared by classical processes (e.g., freeze–thaw and microphase separation) with highly conductive materials (silver nanowires, graphene, etc.) [81, 87]. The self-repair ability of high-strength conductive gels is due to dynamic covalent bonding, which further enhances their mechanical stability. The use of such gels as a flexible electrode in TENGs can greatly improve the working stability and accuracy in extreme environments. In addition, biocompatible gels can be placed in direct contact with human skin or tissue to enable the application of TENGs in medical and health monitoring.

**Table 1:** Advantages and disadvantages of various gels and their sensing applications in TENGs

Types	Roles in TENG	Advantages	Disadvantages	Sensing Applications in TENG
Hydrogel	Electrode [71] Tribolayer [141] Substrate [65]	High conductivity [142] Mechanical flexibility [143] Self-healing [144] Biocompatibility [71]	Solvent loss leads to electrode failure [71] Difficult to balance conductivity and transparency [140] Requires elastomer encapsulation; poor interfacial adhesion [145]	Implantable sensing [146] Health monitoring [147] Motion sensing [87] Tactile sensing [89]
Organogel	Electrode [148] Tribolayer [149]	Frost-resistance [75] Toughness [80] Environmental adaptability [77]	Insufficient conductivity Poor interfacial adhesion [78]	Implantable sensing [76] Motion sensing [148] Smart fabrics [77]
Aerogel	Electrode [150] Tribolayer [69]	Lightweight Compressibility Porous structure Thermal insulation [151]	Limited strain sensing range Electrical properties affected by humidity [88] Insufficient mechanical properties [86]	Gas sensing [152] Foot motion sensing [88] Temperature sensing [94]

Organogels are mechanically more flexible and stable than hydrogels and can maintain good flexibility and stretchability as flexible electrode materials over a wide range of stress and environmental conditions (temperature and humidity variations) [79, 153]. These favorable properties could enable organogel-based TENGs to be used for the long-term monitoring of bending motions. There is a wide range of options for the liquid phase of an organogel and its continuous phase can comprise polar organic solvents and their aqueous mixtures [148], ionic liquids, fats, or oils [154, 155]. Ionogels with excellent ionic conductivities are being investigated as flexible electrodes. Organogels with excellent environmental tolerance, mechanical toughness, and temperature resistance can be developed for TENG applications by replacing or changing their solute properties [73, 83, 156–159], which is important for enhancing the durability and application range of flexible sensors.

Highly porous aerogel materials can be applied to TENGs for high-temperature and gas sensing because of their thermal insulation and breathability [94, 152]. Their porous structure and light weight make aerogels preferred materials for flexible friction layers [69]. Conductive aerogels can be used as electrodes for flexible TENG sensing over a small strain range or as friction layers, which is uniquely advantageous for the miniaturization of TENG sensing devices.

In general, the three-dimensional dynamic network structure of hydrogels and organogels endows them with adjustable conductivity, and their solid-like structure and shape self-repair properties enable them to adapt to various deformation conditions. The special porous structure of aerogels results in their light weight, thermal insulation, and electromagnetic protection properties, which are advantageous for TENG sensor applications. The three types of gels are characterized by their respective advantages and have exhibited extraordinary application value in flexible TENGs. However, the performance limitations of existing gel materials limit their wide application and further development in TENG sensors; for example, elastomer encapsulation is required to avoid solvent loss in hydrogels, the weak adhesion of organogels, and the limited strain range of aerogels. Owing to the complexity and diversity of flexible sensing applications, there are many potential influencing factors in real scenarios that place high demands on the performance of gel materials, especially in terms of their ability to withstand dynamic mechanical deformation, shape adaptability to irregular surfaces, and operational stability in extreme environments. Therefore, there is an urgent need to explore effective solutions for the performance enhancement of hydrogels, organogels, and aerogels based on the application requirements of TENGs for flexible sensing, so as to guide the development and application of gel-based TENGs in flexible sensing.

### Typical Structures and Working Mechanisms of Gel-Based TENGs

The four traditional working modes defined above can be applied to gel-based TENGs. The vertical contact–separation mode relies on the contact–separation motion of the friction layer to generate charge. Owing to its high output efficiency and stability, it is widely used in energy harvesting and self-powered sensing fields. However, the vertical contact–separation mode may not be suitable for the development of small devices and complex multidimensional motion sensors because of the need for a gap between the upper and lower layers and its limitation in generating electricity in a single direction [141]. For the sliding and single-layer modes, although there is no need for set a gap, the wear of the friction layer caused by sliding is not conducive to the long-term use of gel-based triboelectric sensors. Therefore, gel-based TENGs that use these two modes have certain limitations for developing flexible sensors [160]. In contrast, the single-electrode mode has a simple structure and can directly contact human skin. In addition, the other charge-generating surface does not require electrode, allowing greater flexibility, functionality, and applicability of the device [104]. This mode of gel-based TENG combines the advantages of single-electrode and gel materials in terms of both structure and function, making it particularly suitable for use in flexible wearable sensors [71].

**Fig. 3:**
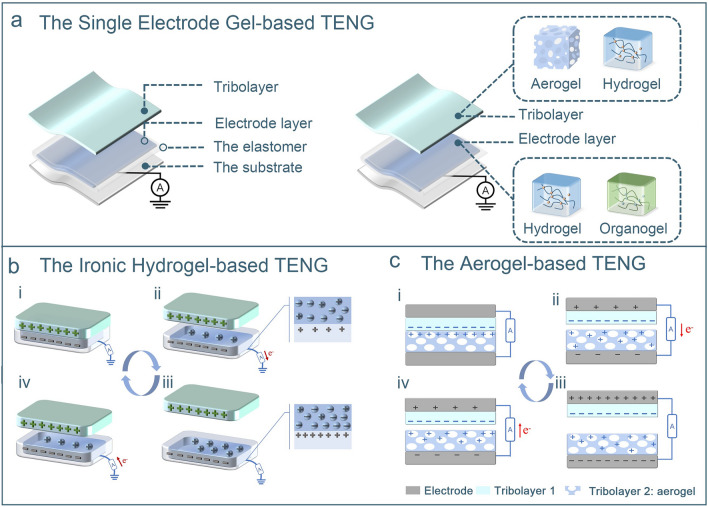
Typical structures and working mechanisms of gel-based TENGs. **a** Structure of single-electrode gel-based TENGs. **b** Working mechanism of ionic hydrogel-based TENGs. **c** Working mechanism of TENGs with aerogel as a tribolayer

In TENG structures, gels are commonly used as charge-exporting layers (electrodes) or charge-generating layers (tribolayers). As shown in Fig. 3a, hydrogels and organogels are frequently utilized as electrodes in TENGs operating in the single-electrode mode. Hydrogel- and organogel-based TENGs often require gel encapsulation within an elastomer, such as Ecoflex [161, 162], PDMS [65, 139, 146, 163], silicone elastomers [97, 164], and polyacrylate elastomers [100]. These elastomers are employed as a single tribolayer for contact charging, whereas materials such as fabric, latex, or skin serve as the other tribolayer [104]. The addition of elastomers significantly mitigates the damage caused by material dehydration, thereby ensuring the stability of the gel structure and the properties of the TENGs.

The working mechanisms of ionic and electronic hydrogels in TENGs differ because of the distinct characteristics of hydrogel conductors. Figure 3b shows a single-electrode TENG with an ionic hydrogel electrode [89]. When the elastomer makes contact with tribolayers exhibiting different dielectric constants, equal magnitude charges of opposite polarity are generated on the surfaces of the elastomer and the tribolayer (Fig. 3b–i). Upon the separation of the two triboelectric layers, the electrostatic charge on the elastomer surface triggers the redistribution of ions within the ionic hydrogel. Positive ions migrate towards the negative charge on the elastomer surface, whereas negative ions accumulate at the hydrogel-wire interface. The connecting wire transports an equivalent number of positive charges owing to interfacial polarization, whereas negative charges flow in the opposite direction towards the grounded end, thereby generating an electrical current signal (Fig. 3b-ii). At the maximum distance between the two tribolayers, all electrostatic charges in the elastomer film are shielded, and no current is generated (Fig. 3b-iii). As depicted in Fig. 3b-iv, when the two tribolayers come into contact again, electrons flow from the ground to the wire. This cyclic contact–separation process produces a continuous alternating current (AC). TENGs with electronic hydrogels as electrodes operate under the same principles as traditional TENGs, relying on electron transfer for power generation [68].

The use of gels as tribolayers in TENGs has received less comprehensive research attention than their use as electrode layers. Nevertheless, existing reports highlight the research value of gel materials in tribolayer designs. Of particular interest are aerogels, which offer distinct advantages owing to their high specific surface area and porosity [31, 84, 88, 92, 151, 165–167]. In the vertical contact–separation mode of aerogel-based TENGs, two tribolayer materials with different dielectric constants are vertically stacked. One of the tribolayers is an aerogel material and metal electrodes are applied to the backsides of both materials (Fig. 3c). This configuration enables the generation of a continuous AC output via contact–separation cycles, similar to traditional TENGs [168]. Notably, the compressibility of the aerogels enhances the deformability of the device, whereas the presence of pores facilitates the storage and transfer of charged materials, thereby effectively increasing the surface charge density of the tribolayers [68, 84]. Consequently, under identical conditions, TENGs with aerogel tribolayers demonstrate superior electrical output compared to those with employing dense tribolayers.

## Optimizing Material Performance of Gel-Based TENGs for Flexible Sensing

As previously indicated, gel-based TENGs have demonstrated significant benefits and application potential in flexible sensing owing to the unique merits of gels, including electrical conductivity and stretchability. However, traditional gels have obvious deficiencies in the aforementioned properties, which makes it difficult to ensure that gel-based TENGs maintain good output performance in complex and variable scenarios. To enhance the sensitivity, stability, and durability of gel-based TENGs for flexible sensing applications, it is necessary to optimize the performance of the gel materials. The conductivity, mechanical properties, and environmental adaptability of gel materials are important factors that affect the sensing performance and durability of flexible triboelectric sensors. This section focuses on the performance optimization of three types of gel-based TENGs that are in high demand for flexible sensing.

### Hydrogel-Based TENGs

Hydrogels are polymers with three-dimensional network structures formed by different mechanisms, such as physical entanglement, electrostatic interactions, and covalent chemical cross-linking using natural or synthetic materials [39]. Based on the differences in crosslinking mechanisms, hydrogels are categorized into physically crosslinked, chemically crosslinked, and physical–chemical hybrid hydrogels [67]. Most TENG studies investigate chemically crosslinked and hybrid hydrogels [169]. The matrix materials used for TENG gels are PAM, PVA, and cellulose [66]. As soft ion conductors, the conductivity, mechanical properties (toughness, strength, and elongation), and self-healing ability of hydrogels should be further optimized [170–172], so as to ensure good mechanical durability and output stability of flexible triboelectric sensors [173]. Further design optimization is expected to yield multifunctional hydrogel-based TENGs with self-cleaning and smart responses, making them suitable for sensing in special environments.

#### Conductivity

Since hydrogels are typically used as electrode layers in the development of flexible triboelectric sensors, their conductivity is the most important material property. However, hydrogels typically have intrinsic conductivities of 10^–5^–10^–1^ S cm^−1^, which is 6–9 orders of magnitude lower than that of metallic materials. This low conductivity renders them unsuitable as flexible triboelectric sensors. Therefore, it is important to increase the conductivity of hydrogel electrodes to enhance the sensing effect of flexible triboelectric sensors. Generally, common strategies to enhance conductivity include the introduction of conductive fillers or dopants [174–177], free ions [178, 179] and conductive polymers into the gel matrix [180–182].

**Fig. 4:**
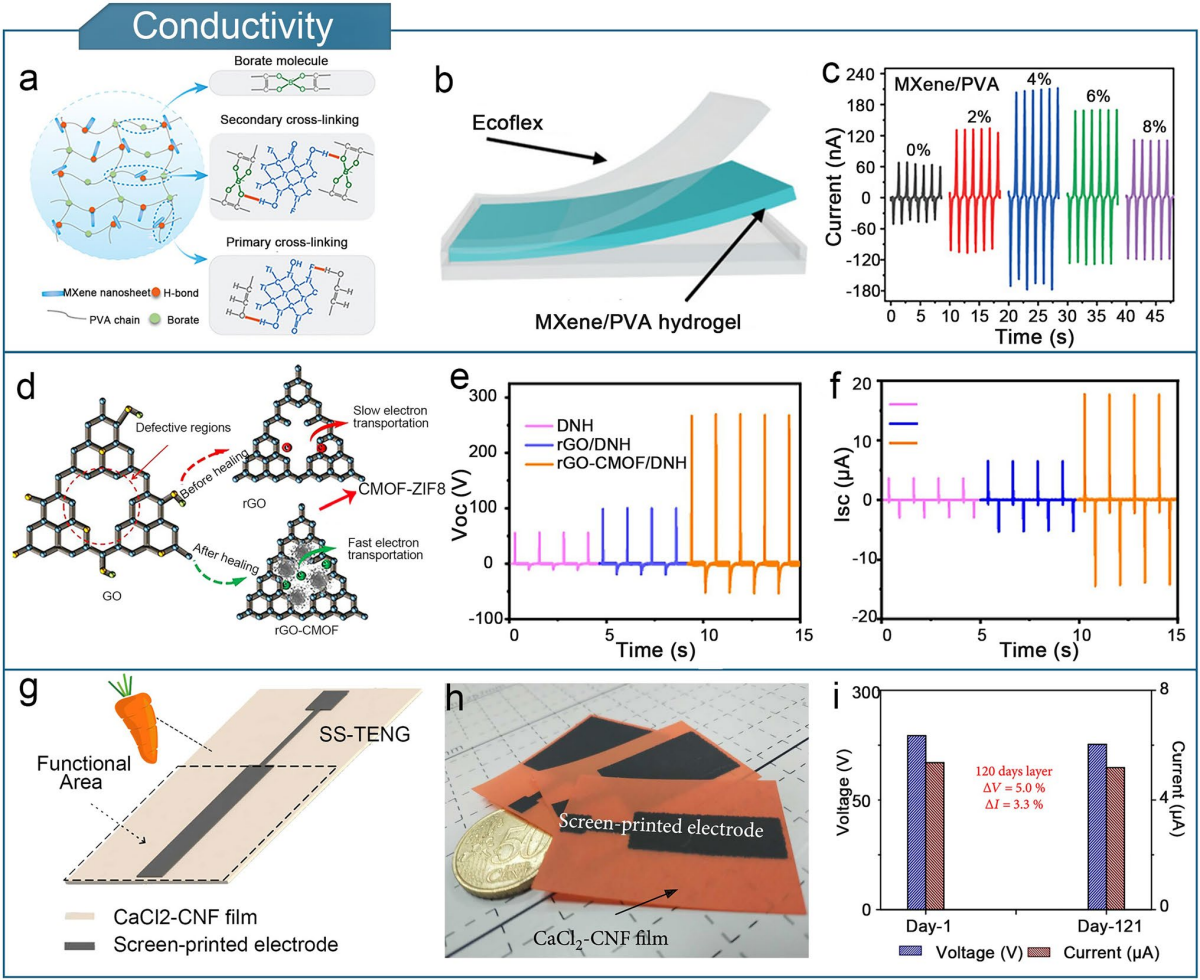
Conductivity optimization of hydrogel-based TENGs. **a** Molecular structure of PVA/MXene hydrogel. **b** PVA/MXene hydrogel-based TENG. **c** Output currents of PVA/MXene hydrogel-based TENG with various MXene doping levels. **a**–**c** Reproduced with permission from Ref. [183], Copyright 2021, Wiley–VCH. **d** Repair mechanism of rGO-CMOF/DNH TENG. **e** Output voltage of rGO-CMOF/DNH TENG. **f** Output current of rGO-CMOF/DNH TENG. **d**–**f** Reproduced with permission from Ref. [142], Copyright 2023, Elsevier. **g** Structure of SOP-TENG. **h** Photograph of SOP-TENG. **i** Effect of aging on the voltage output of SOP-TENG. **g**–**i** Reproduced with permission from Ref. [184], Copyright 2021, American Association for the Advancement of Science

Conductivity enhancement by introducing conductive fillers or dopants is achieved by the generation of conductive transport channels inside the hydrogel, which accelerates ion transport, and increases the conductivity [136, 142, 150, 183]. Figure 4a shows a schematic of the structure of a PVA–MXene hydrogel [183]. MXene nanosheets possess abundant surface functional groups, providing additional hydrogen-bonding sites for the PVA molecular chains and forming primary cross-links. Additionally, borate salts were doped as cross-linkers, promoting secondary crosslinking between MXene and PVA. Under the dual cross-linking of MXene and borate salts, the conductivity and tensile properties of the PVA hydrogel are greatly improved. The hydrogel was encapsulated in an Ecoflex silicone rubber to assemble a single-electrode TENG (Fig. 4b). Based on the streaming vibration potential model, the structure of the MXene nanosheets resembled a microchannel filled with water, facilitating the transport of positive ions in the MXene/PVA hydrogel after frictional charging, thereby enhancing the output performance. The TENG output was enhanced by a factor of four with a doping concentration of 4% MXene nanosheets (Fig. 4c). Furthermore, the conductivity was enhanced by incorporating a carbonized metal–organic framework (CMOF) to repair the defect regions within reduction graphene oxide (rGO), which was used as a dopant for a carboxymethyl cellulose (CMC)/PVA/EG double-network hydrogel (DNH) [142]. The rGO-CMOF/CMC/PVA/EG DNH electrode exhibited an enhanced electron transfer capability, which was attributed to the conductive network formed by CMOF-rGO (Fig. 4d). Compared with pure DNH, the electrical output of the TENG in rGO-CMOF/CMC/PVA/EG DNH assembly showed significant improvement (Fig. 4e, f). Furthermore, a flexible stable output-performance(SOP)-TENG was constructed based on the coupling mechanism of electrostatic induction and ionic conduction [184]. Figures 4g, h show that the SOP-TENG consists of a calcium chloride–cellulose nanofibers (CaCl_2_–CNF) hydrogel film and graphite printed electrodes. Doping with CaCl_2_ confered excellent electrical conductivity to the CNF hydrogel films (made from regenerated carotene). In addition, device aging had little impact on the performance of the SOP-TENG, which maintained an ultrastable electrical output after 120 days (Fig. 4i).

#### Mechanical Performance

To achieve skin adaptability, sensing stability, and device durability, wearable sensing devices have special requirements regarding the mechanical properties of hydrogel materials, particularly their integrity under large tensile deformations. However, the mechanical properties of most traditional hydrogels are still unsatisfactory (strength < 50 kPa, stiffness < 10 kPa, toughness < 10 J m^−2^) [185]. The development of composite materials and energy-dissipation are the primary methods for improving the mechanical properties of hydrogels [186]. In the strength optimization of hydrogel-based TENGs, the main concern is the material properties, such as tensile strength, fracture resistance, and flexibility.

**Fig. 5:**
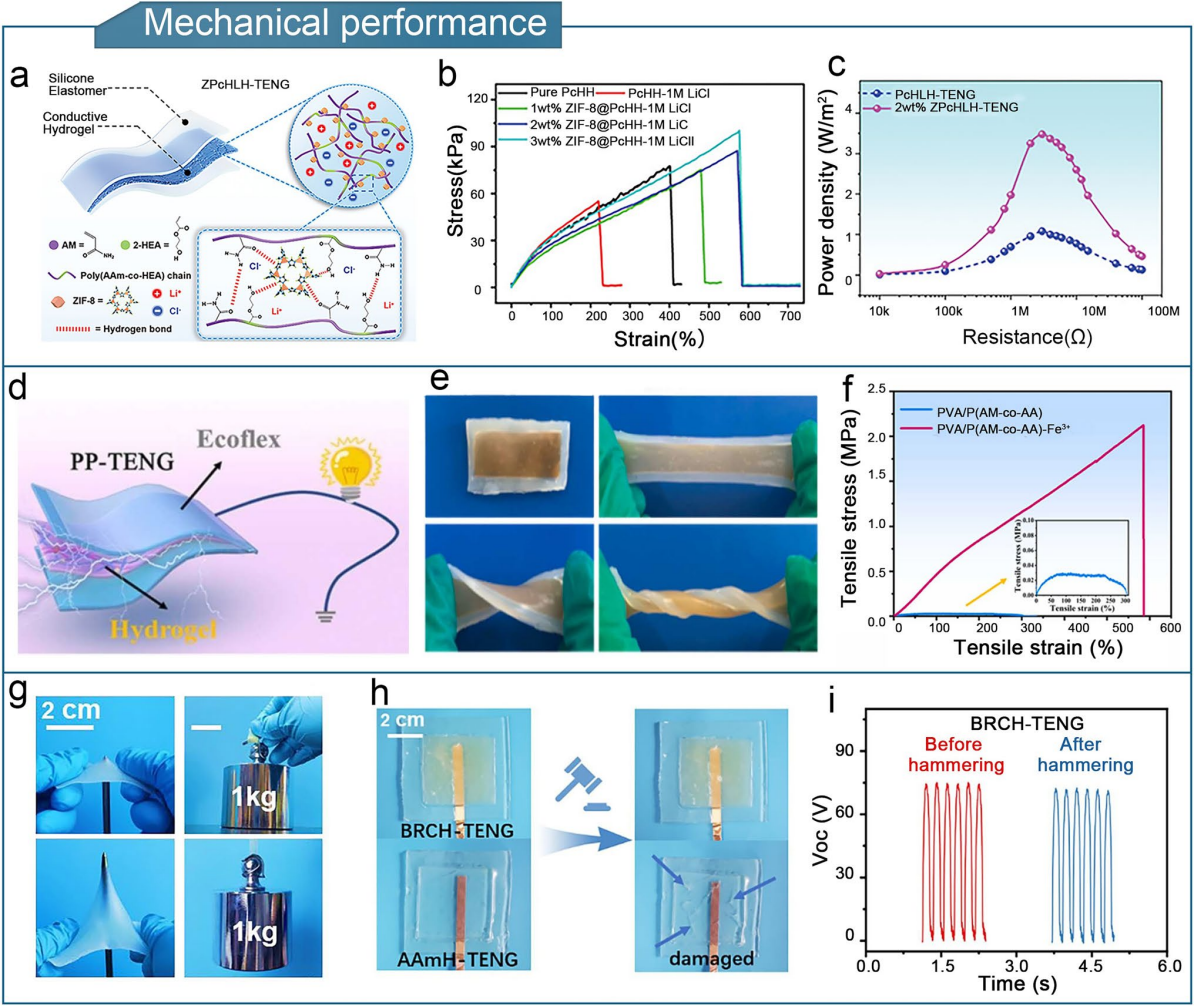
Optimization of the mechanical properties of hydrogel-based TENGs. **a** Molecular structure of ZPcHLH. **b** Tensile stress–strain curves of ZPcHLH prepared with different ZIF-8 doping amounts (0, 1, 2, and 3 wt%). **c** Comparison of power densities of PcHLH-TENG and 2 wt% ZPcHLH-TENGs at various load resistances. **a**–**c** Reproduced with permission from Ref. [99], Copyright 2023, Wiley–VCH. **d** Structure of PP-TENG. **e** Images of PP-TENG in the pristine, tensile, warped, and twisted states. **f** Tensile stress–strain curves of PVA/P(AM-co-AA) and PVA/P(AM-co-AA)-Fe^3+^ hydrogels. **d**–**f** Reproduced with permission from Ref. [87], Copyright 2022, Elsevier. **g** Schematic of a BRCH containing 8 wt% starch being pierced by a sharp steel needle and lifting a 1 kg weight. **h** Schematic of BRCH-TENG and control before and after hammering. **i** Output voltage of BRCH-TENG before and after hammering. **g**–**i** Reproduced with permission from Ref. [143], Copyright 2023, Elsevier

The addition of nanofillers or dopants is a common strategy for enhancing the mechanical strength of hydrogels. In composite materials, the main strengthening mechanism involves the generation, copolymerization, or doping of nanomaterials into the internal polymer network of the gel. Under external loading conditions, nanomaterials resist and disperse high loads, thereby greatly significantly improving their fracture toughness [187, 188]. The zeolitic imidazolate framework-8 (ZIF-8) was used as a nanofiller and incorporated into a lithium chloride (LiCl)-containing PAM-co-hydroxyethyl acrylate (HEA) hydrogel, abbreviated as ZPcHLH [99]. The ZPcHLH was used to assemble a single-electrode triboelectric nanogenerator (ZPcHLH-TENG) (Fig. 5a). The ZIF-8 nanocrystals improve the physical crosslinking of the hydrogel by forming hydrogen bonds with the copolymer chains. Compared with the PAM-co-HEA-LiCl hydrogel (PcHLH) with undoped ZIF-8 nanofillers, the ZPcHLH showed a 2.7-fold enhancement in tensile properties and a strain at break of up to 570% (Fig. 5b). Moreover, the double-layer charge-transfer mode significantly enhanced the performance of the ZPcHLH-TENG, leading to a maximum power density of 3.47 W m^−2^ (Fig. 5c).

The energy-dissipation enhancement strategy is mainly achieved by developing DNHs. In these hydrogels, sacrificial “weak bonds” are introduced into the gel network, which are the first to break under an applied force, thereby consuming a significant amount of energy. Subsequently, the “strong bonds” that maintain the integrity of the gel break, leading to hydrogels demonstrating both high strength and high toughness [189–191]. A DNH comprising borax-crosslinked PVA as the first network and Fe^3+^-crosslinked PAM-polyacrylic acid (PAA) as the second network was proposed [87]. The DNH exhibited strong mechanical properties, with a tensile strain of 590% and tensile stress of 2.1 MPa. The PVA/P(AM-co-AA)-Fe^3+^ hydrogel-based TENG (PP-TENG) demonstrated high sensitivity (gauge factor of 2.3) and output stability (Fig. 5d–f). Moreover, based on the Hofmeister effect, a high-strength breakage-resistant conductive hydrogel (BRCH) was created by solvent substitution [143]. The hydrogel consisted of starch and hydroxyethyl methacrylate (HEMA), and the hydrogel electrode exhibited exceptional puncture and fracture resistances, which were attributed to the bundled starch chains within the hydrogel (Fig. 5g, h). The maximum compressive stress reached 6.83 MPa, which is significantly higher than that of conventional hydrogel electrodes. The hydrogel-based TENG exhibited stable performance in high-impact environments (Fig. 5i), which fundamentally improved its mechanical durability.

#### Self-Healing

High-strength and highly conductive hydrogels are suitable for use in TENGs for flexible sensing applications. However, to expand the range of applications to extremely harsh environments, flexible sensing devices with self-healing capabilities must be developed. Currently, the self-healing of hydrogel-based TENGs mostly relies on dynamic chemical and physical bonding [98, 144, 192–195].

**Fig. 6:**
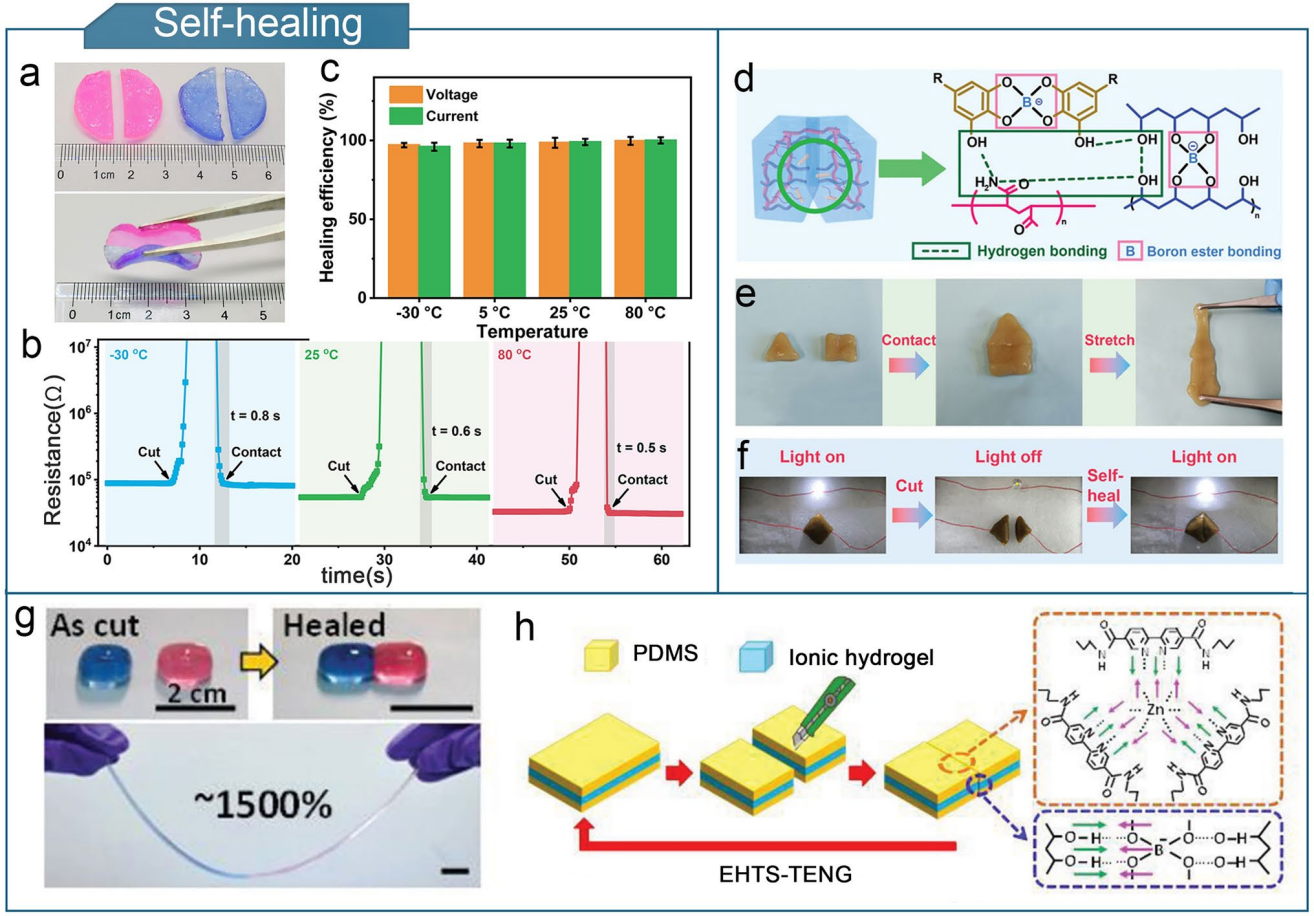
Optimization of self-healing properties for hydrogel-based TENGs. **a** PAM–clay hydrogel self-healing process. **b** Resistance change of PAM–clay hydrogel-based TENG during cutting and healing at different temperatures. **c** Healing efficiency and output stability of PAM–clay hydrogel-based TENG at different temperatures. **a**–**c** Reproduced with permission from Ref. [192], Copyright 2021, Elsevier. **d** Self-healing mechanism of PPC. **e** Photograph of two different shapes of PPC healing into one piece, followed by stretching. **f** Photograph of PPC in a circuit with an LED bulb. **d**–**f** Reproduced with permission from Ref. [98], Copyright 2023, Wiley–VCH. **g** Self-healing behavior of the ionic hydrogel. **h** Self-healing mechanism of EHTS-TENG. **g**, **h** Reproduced with permission from Ref. [194], Copyright 2019, Wiley–VCH

A PAM–clay hydrogel that can heal over a wide temperature range (− 30 to 80 °C) within 1 s was developed [192]. The hydrogel-based TENG maintained a high output over a wide temperature range owing to the antifreeze electrode and ice-phobic triboelectric layer (Fig. 6a–c). A self-healing hydrogel composed of acrylic acid (AA) grafted with gum arabic (GA) was reported [144]. The hydrogel matrix was enriched with multiple dynamic covalent bonds, resulting in self-healing capabilities. A self-healing hydrogel-assembled single-electrode TENG was integrated into a hydrogel touch panel. The panel maintained a fast response, high resolution, and a quick self-repair function even at sub-zero temperatures (− 20 °C) and high tensile states (1600% surface strain). In addition, a novel PVA–PAM/tannic acid-modified cellulose nanocrystalline double network hydrogel (PPC) was developed [98]. Owing to the hydrogen and boron ester bonds, PPC exhibits rapid self-healing ability, can stretch without breaking after 2 min of self-healing, and has excellent electrical conductivity (Fig. 6d–f). The PPC-TENG was used to monitor instantaneous vehicle speeds in intelligent traffic-monitoring systems. Furthermore, an entirely self-healing, transparent, and stretchable ionic hydrogel-based TENG (EHTS-TENG) was presented [194]. At room temperature, this ionic hydrogel self-healed in two minutes and withstood 1500% strain after healing (Fig. 6g). Owing to the dynamic metal–ligand bonding and hydrogen bonding interactions, the EHTS-TENG achieved self-healing without requiring heating and completely healed after 30 min at 900% strain (Fig. 6h). Moreover, even after 500 cycles, the TENG was able to regenerate and continue producing electricity.

#### Other Properties

In addition to the aforementioned performance optimizations, significant advancements have been made in the development of hydrogel-based TENGs in terms of their freezing resistance, self-cleaning, and smart response functions. These advances are important for enhancing the sensing sensitivity and detection range of hydrogel-based TENGs in specific environments [196].

**Fig. 7:**
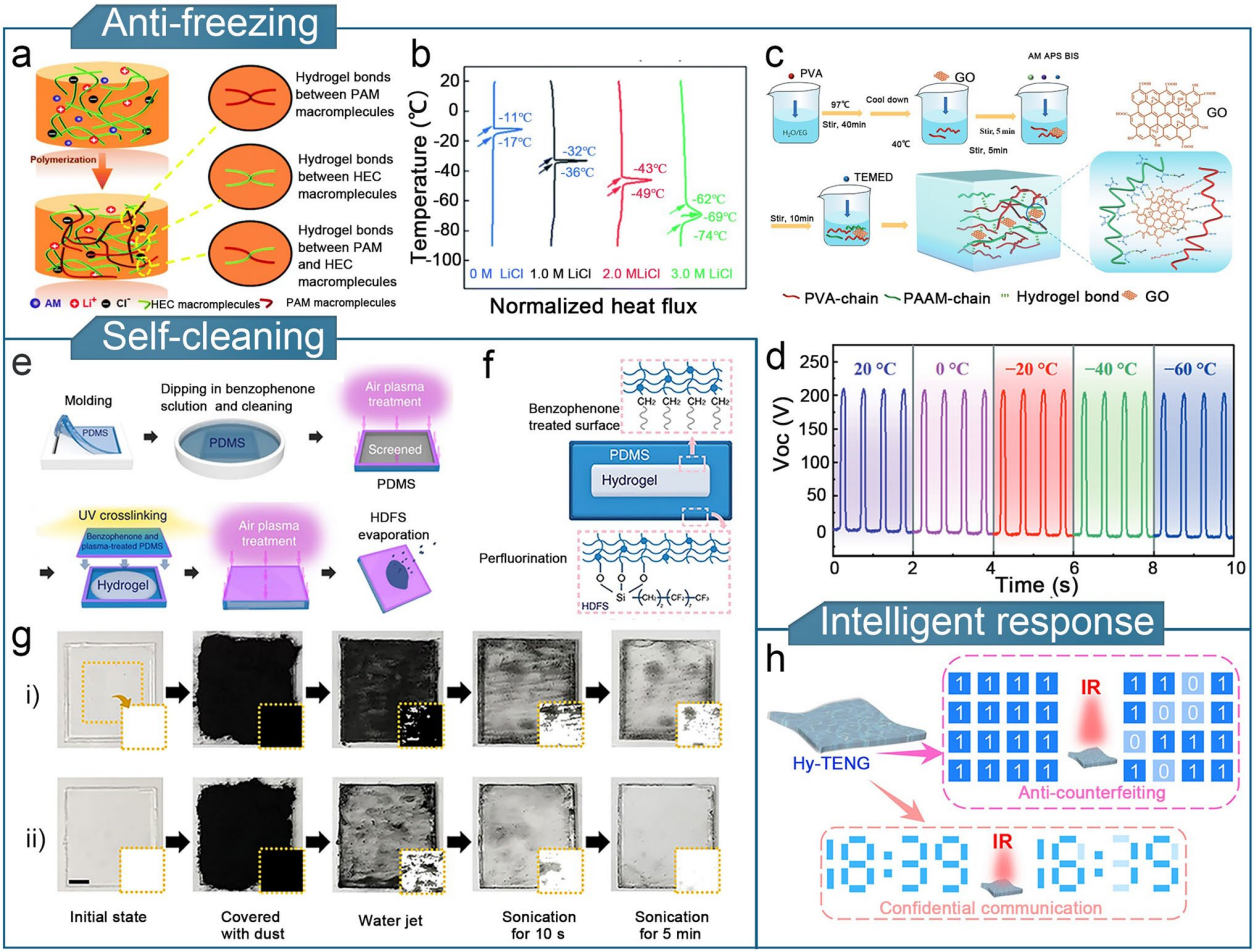
Optimization of other properties of hydrogel-based TENGs. **a** Polymerization process of PAM/HEC/LiCl hydrogels. **b** Antifreeze properties of PAM/HEC/LiCl hydrogels with different LiCl dopant contens. **a**, **b** Reproduced with permission from Ref. [197], Copyright 2020, Royal Society of Chemistry. **c** Preparation of GPPD-hydrogel. **d** Output voltage of GPPD-TENG at different temperatures. **c, d** Reproduced with permission from Ref. [198], Copyright 2022, Tsinghua University Press. **e** Manufacturing process and **f** cross-sectional structure of an ion communicator. **g** Comparison of the self-cleaning effectiveness of an ion communicators (i) without and (ii) with HDFS treatment. **e–g** Reproduced with permission from Ref. [139], Copyright 2018, Springer Nature. **h** Schematic diagram of Hy-TENG used for smart response. Reproduced with permission from Ref. [203], Copyright 2023, Elsevier

As shown in Fig. 7a, PAM/HEC/LiCl hydrogels were synthesized through the one-step free radical polymerization of an acrylamide monomer (AM) in aqueous hydroxyethyl cellulose (HEC) solution doped with lithium chloride (LiCl) [197]. By adjusting the amount of LiCl added, the hydrogel could be prevented from freezing at − 69 °C (Fig. 7b). Furthermore, HEC introduces hydrogen bonding interactions, which enhance the mechanical properties and water retention of the hydrogel. The TENG assembled with the PAM/HEC/LiCl hydrogel operated efficiently at extremely low temperatures. Furthermore, graphene oxide (GO) was introduced into a PVA–PAM–DNH (GPPD hydrogel) (Fig. 7c) [198]. The GPPD hydrogel demonstrated excellent resistance to low temperatures and an ultrahigh tensile strength (2000%). GPPD-based TENG maintained consistently high performance at − 60 °C, highlighting its durability in extreme environments (Fig. 7d).

Furthermore, gel materials with self-cleaning and antifouling properties must be designed to develop gel electrode surfaces that are resistant to detachment and contamination by continuous frictional contact. A hydrogel ionic conductor chemically bonded to PDMS and PAM/LiCl was demonstrated [139]. HDFS ((heptadecafluoro-1, 1, 2, 2-tetrahydrodecyl) trichlorosilane) was introduced into the TENG (Fig. 7e, f). The self-cleaning capability of the TENG was enhanced by HDFS, and its adhesion to the substrate was improved by PDMS. The hydrogel-based TENG design achieved both self-cleaning and antifouling properties (Fig. 7g), offering a new approach for enhancing the mechanical durability of the device.

Hydrogel materials with responsivity to changes in external factors such as the pH, temperature, light, and heat, are valuable for soft robotics applications [199–202]. Similarly, the development of hydrogel-based TENGs with intelligent response behaviors is also a focus for flexible sensing. Hy-TENG based on a PAM-agar-NaBO-tannin-modified black phosphorus composite hydrogel electrode material was developed [203]. The photothermal properties of black phosphorus enables the temperature-responsive behavior of the hydrogel. Hy-TENG decodes temperature-sensitive modes using infrared light, thereby contributing to the advancement of intelligent sensors (Fig. 7h).

### Organogel-Based TENGs

Similar to hydrogels, organogels are frequently used electrode materials. Organogels are colloids, supramolecules, or polymer three-dimensional networks filled with organic liquids [39, 204]. Organogels exhibit greater solute selectivity than hydrogels. Gels composed of polar organic solvents mixed with water [148], ionic liquids, fats, and oils all fall under the category as organogels [154, 155]. Among them, the most studied TENGs are alcohol gels [205], ionogels [206], and organohydrogels [140, 207, 208]. Alcohol gels are particularly useful because of their tunable freezing and boiling points, which allow them to maintain a stable performance over a wide temperature range [209], thereby improving the temperature adaptability of flexible TENGs. Ionogels composed of ionic liquids exhibit high ionic conductivity [210], freezing resistance [211], and thermal and chemical stability, providing good environmental tolerance in TENGs [212–214]. Organohydrogels can partially overcome typical disadvantages of hydrogels, such as water loss and weak conductivity. Organohydrogels are often applied in flexible TENGs as electrode materials [148, 215]. Further optimization of the mechanical toughness, temperature resistance, and environmental adaptability of organogels can enhance the mechanical durability and sensing sensitivity of flexible triboelectric-based sensors [39].

#### Mechanical Toughness

Flexible sensors often need to withstand certain stresses and deformations, particularly under unconventional deformation conditions such as bending, stretching, and twisting. Designing materials with high mechanical toughness can help flexible sensors better adapt to complex environmental changes, to help prevent premature device failure and enhance their stability and lifespan. Similar to mechanically enhanced DNHs, a common strategy for achieving high toughness in organogels is to include energy-dissipation mechanisms. Some common methods include the introduction of sacrificial networks [79, 80], phase separation effects [81], polymer crystallization, and ion–dipole interactions [82, 83].

**Fig. 8:**
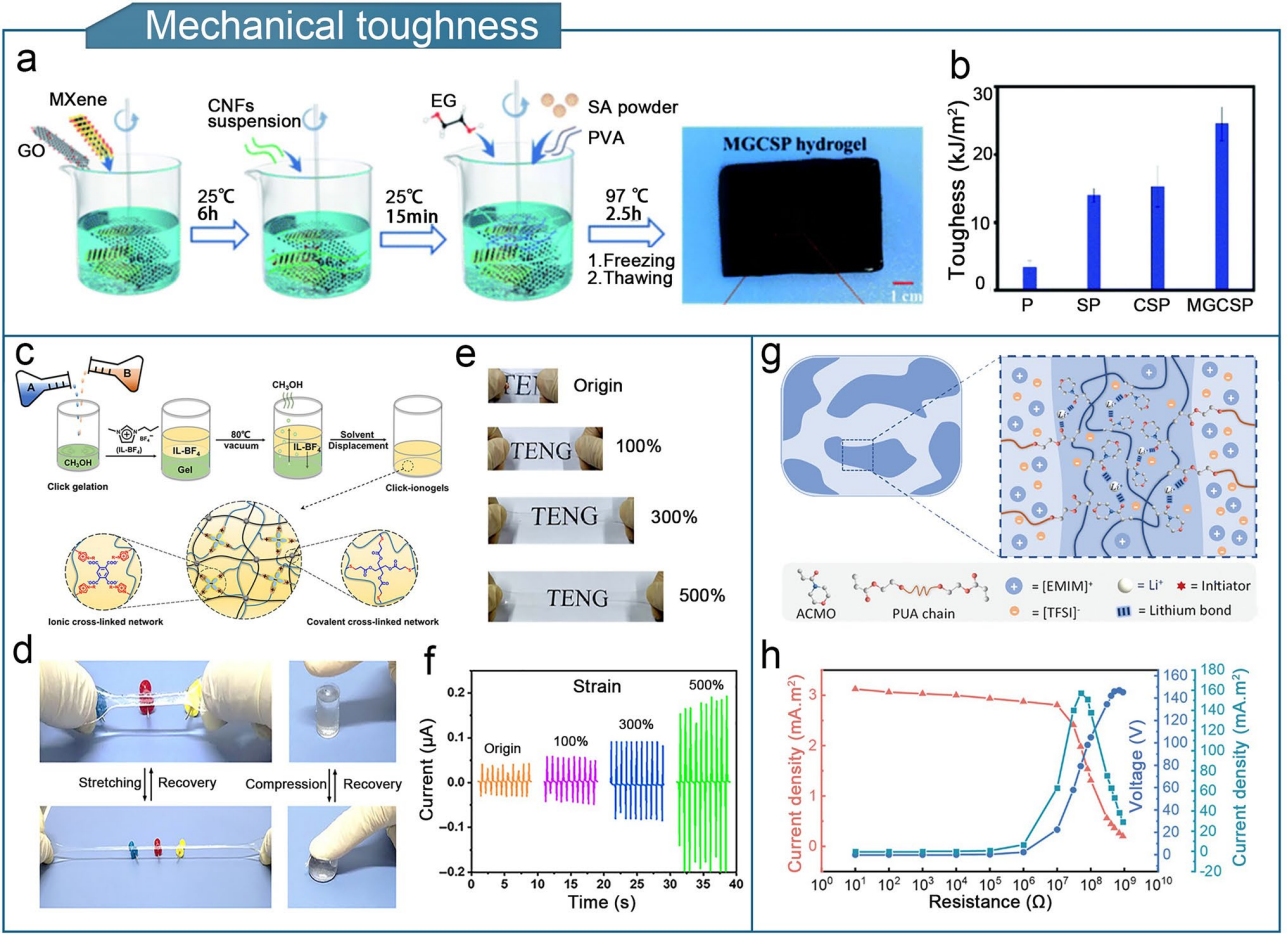
Optimization of mechanical toughness of organogel-based TENGs. **a** Preparation of MX-GO/CNF/SA/PVA organohydrogels. **b** Comparison of the toughness of MX-GO/CNF/SA/PVA with those of other organohydrogels. **a**, **b** Reproduced with permission from Ref. [80], Copyright 2022, Royal Society of Chemistry. **c** Preparation and structure of a thiol-enclosed ionogel. **d** Photographs of thiol-enclosed ionogel in stretching and compression. **e** Photographs of ionogel-based TENG under stretching. **f** Output currents of ionogel-based TENG in different stretching states. **c**–**f** Reproduced with permission from Ref. [79], Copyright 2019, American Association for the Advancement of Science. **g** Microphase separation structure of the ionogel. **h** Output electrical signals of the ionogel-based TENG at different external loading resistances. **g**, **h** Reproduced with permission from Ref. [81], Copyright 2023, Wiley–VCH

The fabrication of triple-network crosslinked structures can enhance the toughness of organogels. Figure 8a illustrates the doping of MXene–GO nanocomposites and ethylene glycol solvent into CNF/sodium alginate (SA)/PVA triple-network organohydrogels for the preparation of MX-GO/CNF/SA/PVA organohydrogels [80]. The large number of hydrogen bonds formed by CNF, SA, and PVA significantly enhances the mechanical properties of MX-GO/CNF/SA/PVA organohydrogels, and the toughness reached 24.5 kJ m^−2^, which is 7.2 times higher than that of the pure hydrogel (Fig. 8b). An MX-GO/CNF/SA/PVA organohydrogel-based TENG demonstrated an exceptional electrical output and sensing sensitivity, with a gauge factor of 2.77. Additionally, researchers have used thiol-alkene click chemistry to create ionogels based on ionic liquids by forming a sacrificial network with poly (1-butyl-3-vinyl imidazolium fluoroborate) and benzene tetracarboxylic acid (BTCA) [79]. The ionogel-based TENGs demonstrated excellent sensitivity for detecting and monitoring finger-flexion movements, and the ionogels demonstrated exceptional mechanical toughness and elasticity even after 10,000 fatigue cycles. The TENG exhibited stable electrical output over a wide temperature range, from − 75 to 340 °C. The output current of the TENG was 0.05 μA in its strain-free pristine state, which increased to 0.2 μA after being stretched to a strain of 500% (Fig. 8c–f).

Another useful strategy for increasing the mechanical toughness of organogels is microphase separation [81]. Figure 8g shows that lithium bonds were formed through the interaction of lithium salts and carbonyl groups, leading to microphase separation, i.e., the formation of distinct rigid and soft regions in the ionogels. The soft zone provides high ionic conductivity and stretchability while the rigid region provides exceptional toughness and durability. When 70 wt% of ionic liquid and 10 wt% of lithium bis(trifluoromethane)sulfonimide (LiTFSI) were added (referred to as IG_70%–10%_), the resulting ionic conductivity reached 2.18 mS cm^−1^, while the tensile strength and elongation at break were 2.29 MPa and 1062%, respectively. These values are noticeably higher than those of other ionogels. The ionogel-based TENG showed a stable power output at high strain for extended periods, achieving a maximum power density of 157.1 mW m^−2^ (Fig. 8h). Additionally, supertough and superstretchable ionogels can be created by combining robust polymer crystallization with weak ionic dipolar interactions, it is possible to create super-tough and super-stretchy ionogels [83]. The highly crystalline region dissipates energy during stress–strain processes, whereas the ionic dipole interaction of amorphous polymer chains with ionic liquids enhances the stretchability and elasticity of the gel material. The ionic gel-based TENG (with a single electrode) demonstrated favorable electrical output properties.

#### Temperature Tolerance

Compared with hydrogels, organogels exhibit superior temperature tolerance. Substances such as glycerol, ethylene glycol, lithium bromide, and proline are frequently added [216–219].

**Fig. 9:**
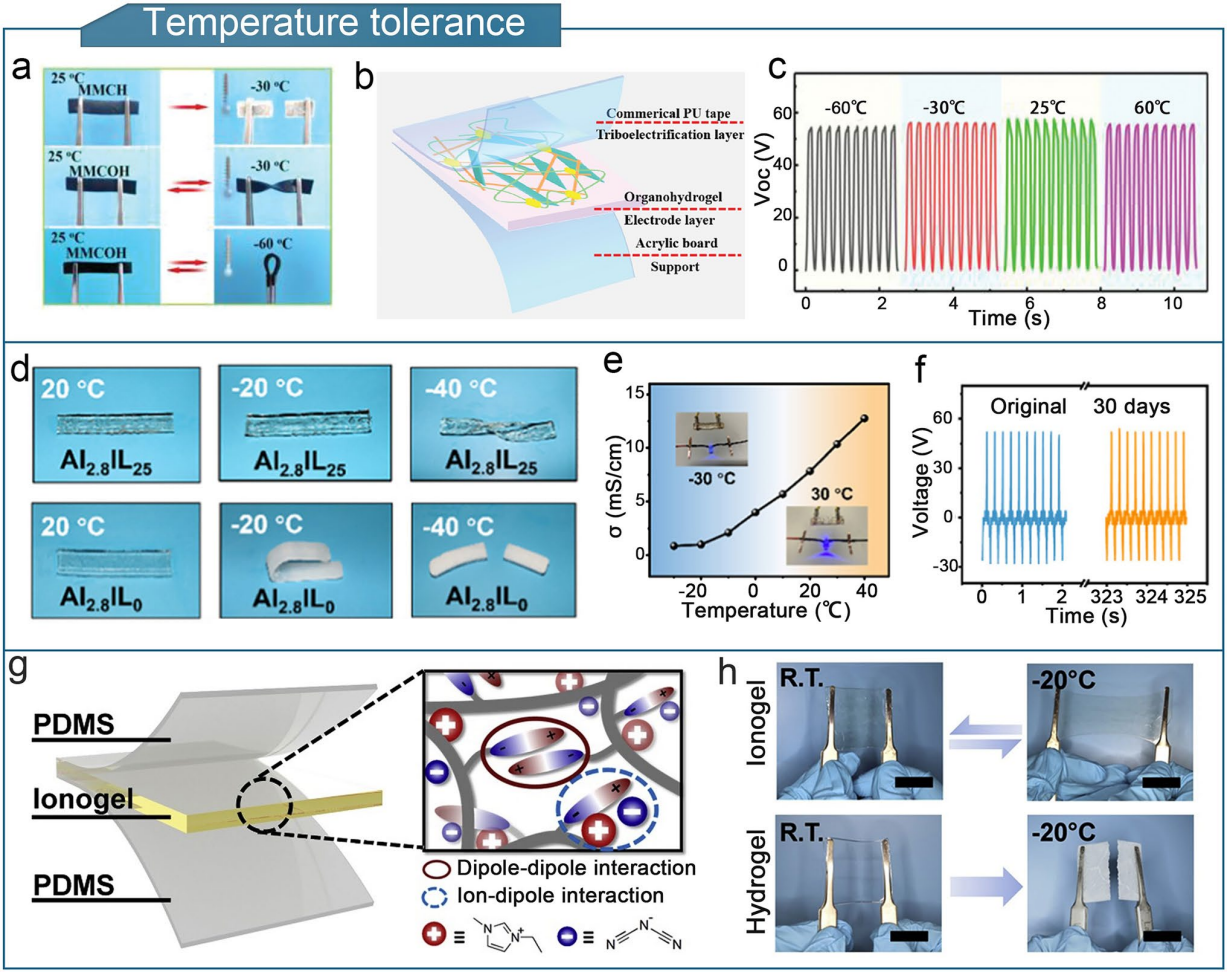
Optimization of the temperature-tolerance of organogel-based TENGs. **a** Freezing resistance of MMCOH. **b** Composition of MMCOH-TENG. **c** Eelectrical output of MMCOH-TENG at − 60 to 60 °C. **a**–**c** Reproduced with permission from Ref. [75], Copyright 2021, Wiley–VCH. **d** Comparison of freezing resistance of organogels with and without ionic liquids. **e** Conductivity of ionic liquid gels from − 30 to 30 °C. **f** Output voltage change of ionic liquid gel-based TENG after 30 d of exposure to air. **d**–**f** Reproduced with permission from Ref. [221], Copyright 2022, American Chemical Society. **g** Structure of ionogel-based TENG containing [EMI][DCA] ionic liquids. **h** Comparison of the low-temperature tolerances of an ionogel-based TENG and typical hydrogel-based TENG. **g**, **h** Reproduced with permission from Ref. [222], Copyright 2019, Elsevier

Solvent replacement is a common and helpful method for improving the overall efficiency of gel TENGs [75, 100, 192, 198, 220]. For example, researchers prepared a PAM/montmorillonite/carbon nanotube (CNT) organohydrogel (MMCOH) using solvent substitution [75]. The MMCOH was used as a tensile electrode (500% strain) in TENGs, which remained environmentally stable at temperatures of − 60 to 60 °C for 30 days (Fig. 9a–c). A self-polymerized multifunctional organogel ionic conductor (MOIC) was constructed using a binary solvent of ethylene glycol and water [153]. The MOIC exhibited ultra-stretchability (9000%), resistance to drying and freezing (− 30 °C), and maintained high mechanical stability after 1800 cycles of loading and unloading at 600% strain. Metal–ligand bonding (Al^3+^) and polymer network cross-linking were used to create an ionic liquid gel based on an ionic-liquid/water binary solvent [221]. The ionogel showed superior resistance to freezing compared to an ionic hydrogel without an ionic liquid. It remained transparent and flexible even at − 40 °C and powered a blue light-emitting device from − 30 to 40 °C (Fig. 9d, e). Owing to the anti-freezing and anti-drying properties of the ionic hydrogel, the TENG assembled with the ionic liquid gel as an electrode maintained a stable output voltage after exposed to air for 30 d (Fig. 9f), demonstrating its excellent temperature tolerance. Furthermore, ion–dipole interactions can be utilized to obtain temperature-tolerant organogels. For example, ionogels containing 1-ethyl-3-methylimidazolyl dicyandiamide ([EMI][DCA]) were synthesized in a single step by the in situ photopolymerization of 3-dimethylammonium (methacryloyloxyethyl) propane sulfonate (DMAPS) and AA, using ammonium persulfate as the photoinitiator (Fig. 9g) [222]. The TENG prepared using the ionogel demonstrated long-term steady electrical output from − 20 to 100 °C because of the strong freezing resistance imparted by the ionic–dipole interactions between the ionic liquid and DMAPS that prevent [EMI][DCA] from crystallizing (Fig. 9h).

#### Other Properties

**Fig. 10:**
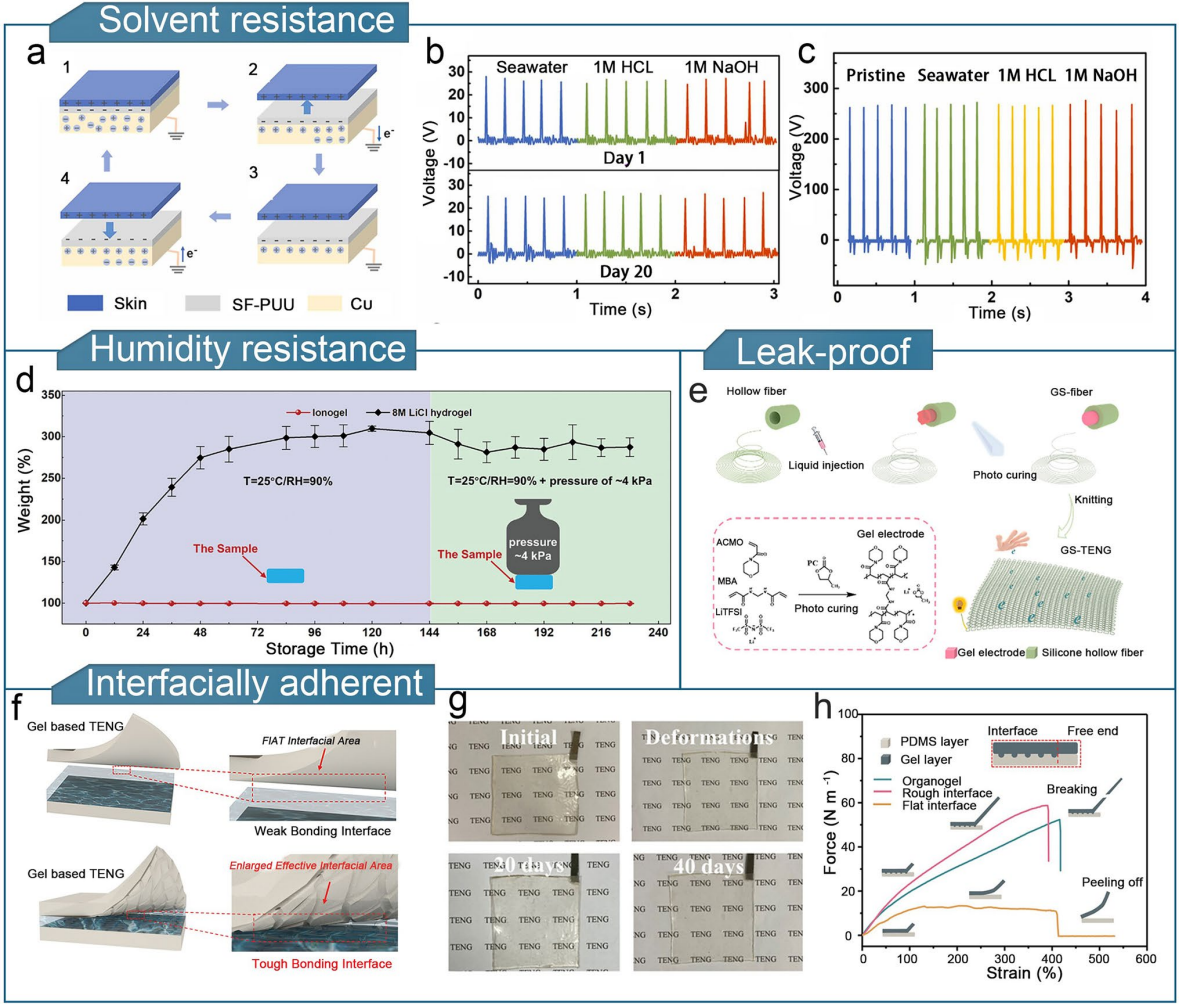
Optimization of other properties of organogel-based TENGs. **a** Structure of FSI-TENG. **b** Open-circuit voltages of FSI-TENG immersed in seawater solution, 1 M hydrochloric acid solution, and 1 M sodium hydroxide solution on days 1 and 20. **c** Comparison of open-circuit voltages of FSI-TENG after immersion, rinsing, and drying. **a**–**c** Reproduced with permission [225]. Copyright 2023, Elsevier. **d** Schematic of the change in weight of the ionogel over time under a stress of ~ 4 kPa and a relative humidity of 90%. Reproduced with permission from Ref. [157], Copyright 2021, Wiley–VCH. **e** Preparation process of GS-TENG. Reproduced with permission from Ref. [77], Copyright 2021, Elsevier. **f** Comparison of smooth and rough gel/elastomer interfaces. **g** Stress–strain curves of organogel/PDMS hybrids with rough and smooth interfaces. **h** Images of rough-interface organogel-based TENG in its initial state, after deformation, and after 20 and 40 d of storage. **f–h** Reproduced with permission from Ref. [78], Copyright 2023, Wiley–VCH

To expand their application potential, flexible sensing devices that can operate in complex liquid environments and under high-humidity conditions are required. For example, in the context of human motion monitoring, the high-humidity and high-salt environment caused by excessive sweating can have a detrimental effect on the sensing performance of wearable TENGs [223]. Encapsulation methods are commonly used to protect gel electrodes from harsh environmental conditions such as acidity, alkalinity, and saltwater. However, the inevitable damage caused by encapsulation rupture can lead to sensing failures [224]. To address this, self-healing fluorinated poly(urethane urea) (SF-PUU) using isocyanate-terminated PDMS was developed. SF-PUU was assembled with self-healing ionogels to form a sandwich structure, creating a fully self-healing triboelectric nanogenerator (FSI-TENG) that could withstand corrosion from 1 M hydrochloric acid, sodium hydroxide solution, and seawater. Owing to the self-healing properties of both the encapsulation layer and ionogel, the FSI-TENG exhibited good reliability and durability (Fig. 10a–c) [225]. Additionally, hydrophobic organogels enhance the moisture resistance of flexible triboelectric sensors, thereby enabling steady output humid conditions. This enhances the stability, accuracy, and durability of the sensor [149, 226]. Poly (ethylene glycol methyl ether acrylate) (PMEA) and poly (isobornyl acrylate) (PIBA) were used to fabricate a highly hydrophobic ionogel containing the ionic-liquid 1-ethyl-3-methylimidazolium bis(trifluoromethylsulfonyl)imide ([C_2_mim][NTf_2_]) [157]. Owing to the high hydrophobicity, high ionic conductivity, and low viscosity of the [C_2_mim][NTf_2_] ionic liquids, the ionogels exhibited a wide operating temperature range (− 60 to 200 °C) and strong interfacial adhesion to elastomers. In addition, the ionogel resisted moisture absorption in high relative humidity environments (25 °C/90% RH) and retained its liquid components under prolonged mechanical loading (Fig. 10d). TENGs developed using ionogels can be fabricated into iono-skins that can simultaneously sense temperature, deformation, and pressure changes, thereby demonstrating excellent environmental adaptability.

To tackle the problem of cracking or leaking in metal or liquid electrodes, researchers have prepared leak-proof gel electrode-based fiber (GS-fiber) by photocrosslinking organogel in transparent organosilicon hollow fiber [77]. The GS-fibers had a gel electrode/silicon core/shell structure and were woven into TENG textiles to detect human motion (Fig. 10e). The flexible but solid organogel electrode avoids issues such as cracking and leakage that are typical limitations of metal and liquid electrodes, respectively, thereby offering an effective solution for the implementation of TENGs in textiles. Furthermore, enhancing the bonding strength between the gel and elastomer is beneficial for improving environmental durability [78, 102]. The precured gel solution was poured onto the rough and smooth surfaces of the PDMS layers. Compared to a smooth PDMS layer, the gel/elastomer interface had a higher surface area because of the in-situ curing of the gel solution poured onto the rough PDMS layer. The TENG with the rough interface did not exhibit significant delamination after various deformations. The device structure remained intact, and the output electrical properties remained stable after 40 d of storage (Fig. 10f–h).

### Aerogel-Based TENGs

Aerogels are ultra-lightweight porous solid scaffolds with a very low density (0.003–0.15 kg cm^−3^), high specific surface area, and high porosity (> 99%) [227]. The porous structure of the aerogels enables good compressibility [228, 229], thermal insulation [74, 230], and electromagnetic shielding properties [231–233]. The high specific surface area and porosity enables aerogels to store and transfer numerous charges, resulting in a high surface charge density. The multi-stage porous structure enables the aerogel to effectively block air convection and reduce heat radiation and conduction, resulting in the superior thermal insulation performance, which meets TENG’s sensing needs in high-temperature environments. Similarly, with the advantages of a lightweight and porous structure, aerogel has certain compressible resilience and electromagnetic shielding performance, which provides a reliable choice for gel-based TENG to broaden the application scenarios. In order to meet the demands of aerogel-based TENG for flexible sensing applications, the relevant properties of aerogels need to be further optimized [234, 235]. In this section, we discuss previous research on enhancing the output performance, thermal insulation, electromagnetic shielding, and strength of aerogel-based TENGs for flexible sensing applications.

#### Dielectric-Enhancement

The dielectric constant describes the capacity of triboelectric materials to produce and retain triboelectric charges in TENGs [236]. Dielectric modulation has proven to be highly effective in enhancing the triboelectric output of aerogel-based TENGs [237]. High-output dielectric-enhanced aerogel-based TENGs can be achieved by adjusting the porosity and filler doping [238].

**Fig. 11:**
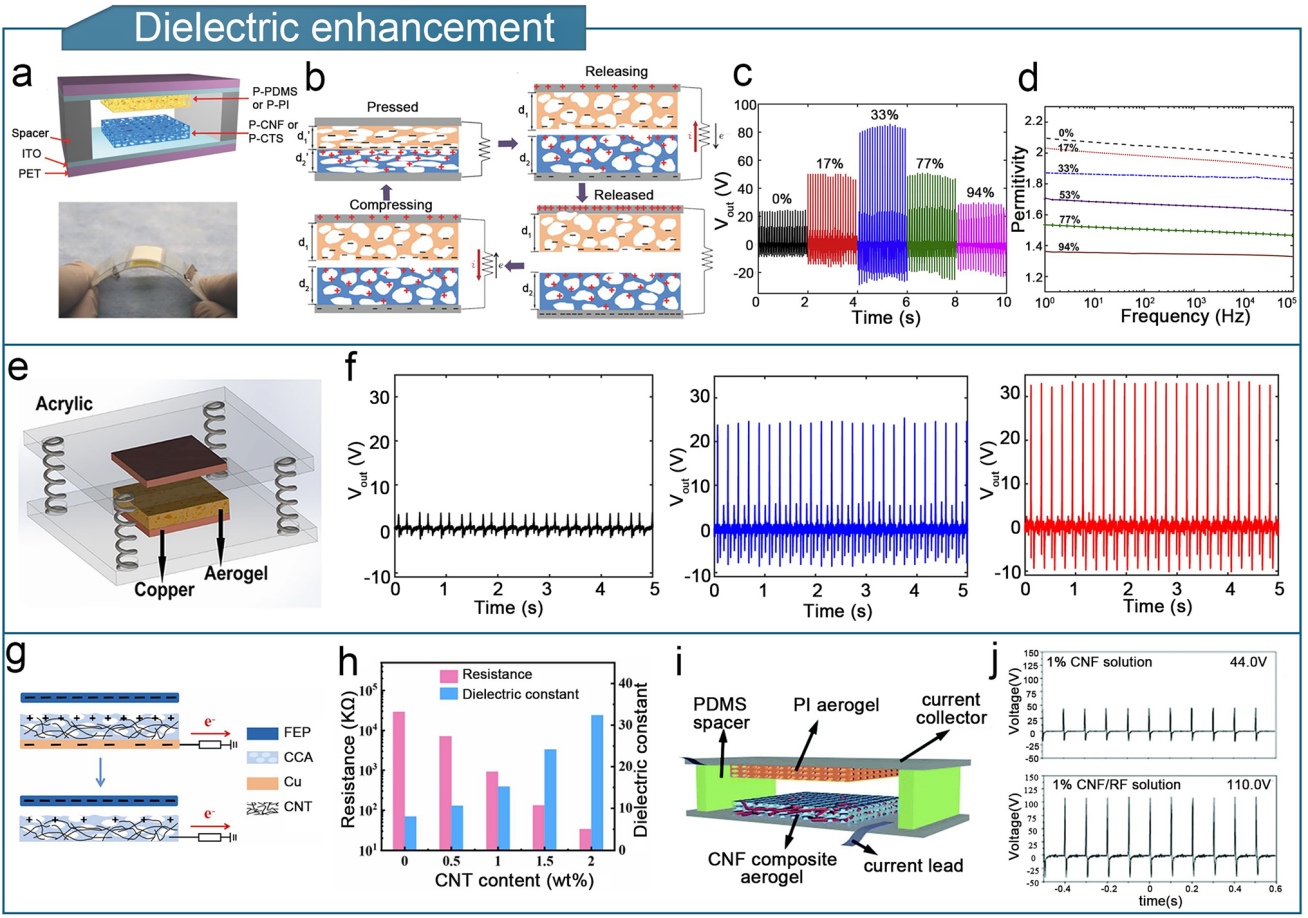
Optimization of dielectric properties of aerogel-based TENGs. **a** Structure and physical diagram of porous chitosan aerogel-based TENG. **b** Working principle of porous chitosan aerogel-based TENG. **a**, **b** Reproduced with permission from Ref. [84], Copyright 2018, Wiley–VCH. **c** Different openings of PU aerogel-TENGs with different output voltage. **d** Effect of open porosity on the dielectric constant of PU aerogel films. **c**, **d** Reproduced with permission from Ref. [73], Copyright 2019, Elsevier. **e** Structure of PI aerogel-based TENG. **f** Comparison of output voltages of PI aerogel-based TENG aerogel base at 0%, 40%, and 50% porosity, respectively. **e**, **f** Reproduced with permission from Ref. [167], Copyright 2019, Springer Nature. **g** Structure of CCA-TENG. **h** Voltage and dielectric constant of CCA-TENG at different external load resistances. **g**, **h** Reproduced with permission from Ref. [88], Copyright 2023, Elsevier. **i** TENG assembled with PI aerogel and CNF composite aerogel. **j** Output voltage of CNF aerogel-based TENG versus CNF/RF aerogel-based TENG. **i**, **j** Reproduced with permission from Ref. [239], Copyright 2018, Royal Society of Chemistry

The porous structure of the aerogel enables it to trap extra charges and facilitate the transport of free ions, effectively increasing the surface charge density. Figure 11a shows a porous aerogel-based TENG consisting of a cellulose nanofiber (CNF) or chitosan (CTS) aerogel film as the tribo-positive layer and a PDMS or polyimide (PI) aerogel film as the tribo-negative layer [84]. Figure 11b shows that the presence of pores enables the aerogel-based tribolayer to store and transfer a large amount of charged material. The power density of the porous chitosan aerogel-based TENG was 11 times higher than that a dense film-based TENG. However, the dielectric enhancement does not always scale with increasing porosity. The triboelectric performance of a polyurethane (PU) aerogel-based TENG gradually increased with increasing porosity up to 33%, after which point the dielectric constant decreased because of the high amount of air inside the aerogel at higher porosities (Fig. 11c, d) [73]. Similarly, Fig. 11e shows a polyethyleneimine aerogel-based TENG [167]. As the open porosity of the aerogel increases from 0 to 50%, the electrical output increases (Fig. 11f), followed by a reduction in the dielectric constant and output when the open porosity exceeds 50% due to an excessive amount of air inside the material and a reduction in the number of polarizable groups per unit volume.

In addition, researchers optimized dielectric-enhanced aerogel-based TENGs by adding fillers that are synergistic with their porous structures [88, 239, 240]. Figure 11g shows a single-electrode cellulose-CNT aerogel-based TENG (CCA-TENG) [88]. The cellulose aerogel doped with CNT exhibited a higher dielectric constant than the pure cellulose aerogel (Fig. 11h), and the CCA-TENG exhibits a power density of 1237 mW m^−2^. Owing to the high density and biodegradability of cellulose, the CCA-TENG exhibited high humidity stability, long-lasting performance, and high cycling efficiency. The performance remained unchanged after 64,800 cycles over 12 months, and 91.04% of its initial output was maintained after cycling. The triboelectric properties of CNF aerogels were enhanced by incorporating silica fibers, human hair, and rabbit fur (RF) into cellulose nanofiber aerogels [239]. The output of the CNF/RF composite aerogel-based TENG was significantly enhanced, yielding an output voltage of 110.0 V, which was substantially higher than that of the pure CNF-aerogel-based TENG (Fig. 11i, j).

#### Thermal Insulation

Temperature variations in the surrounding environment can interfere with the sensing results of flexible sensors. Therefore, it is necessary to identify triboelectric materials with thermally insulating properties to enable flexible TENGs that are resistant to the effects of temperature drift and heat conduction [241]. Aerogels have excellent thermal insulation properties, with a thermal conductivity of 0.012–0.024 W m^−1^ K^−1^, which is 2–3 orders of magnitude lower than conventional insulation materials. The multilayered fractal pore structure of the aerogel effectively prevents air convection, minimizes thermal radiation, and reduces thermal conduction, leading to superior insulating properties [242–244]. Optimizing thermal insulation improves the high-temperature adaptability of the flexible triboelectric sensor while maintaining its sensitivity and sensing threshold.

**Fig. 12:**
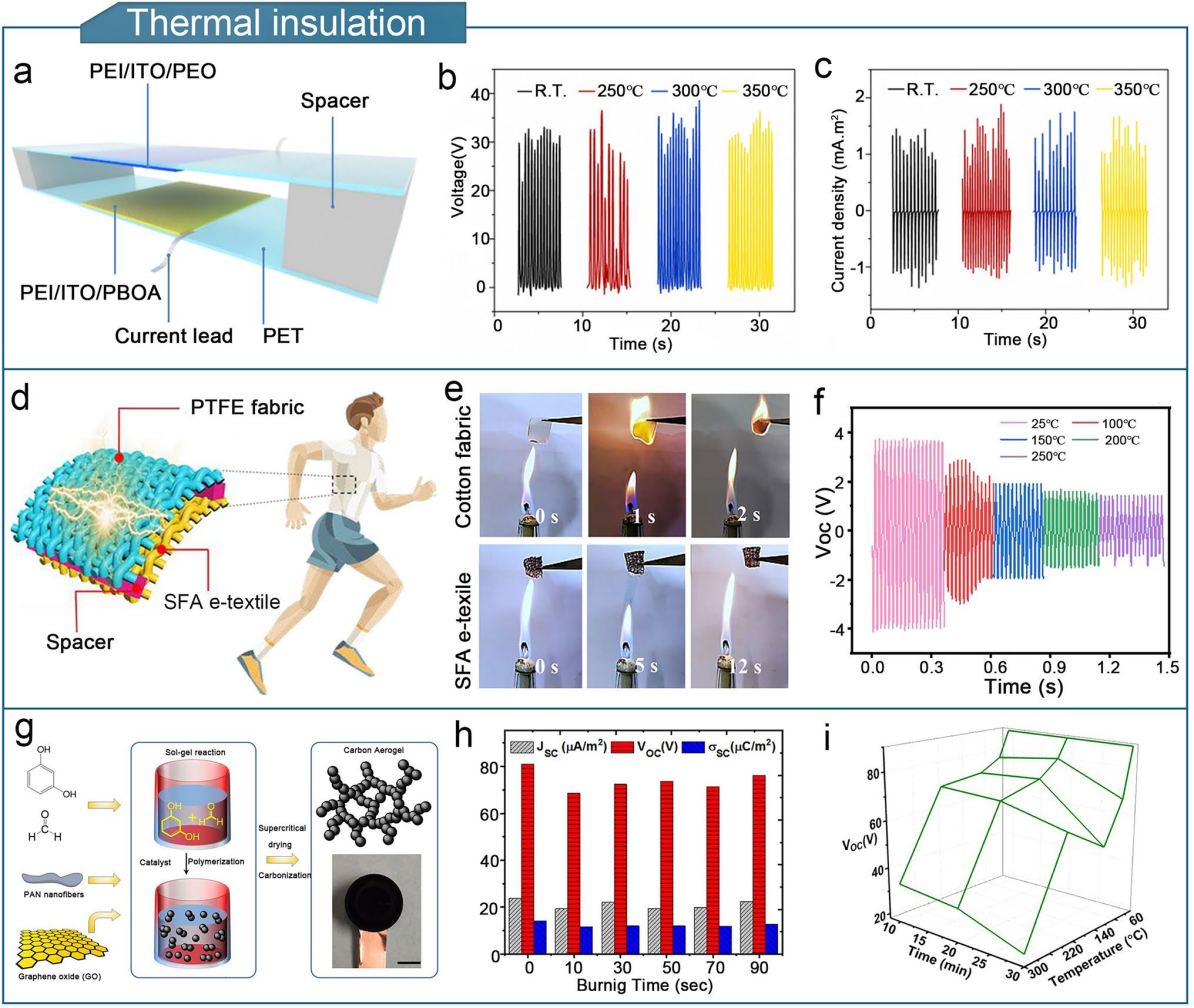
Optimization of thermal insulation properties of aerogel-based TENGs. **a** Structure of PBOA/PEO-TENG. **b** Voltage and **c** current density of PBOA/PEO TENG at different temperatures. **a**–**c** Reproduced with permission from Ref. [151], Copyright 2019, Elsevier. **d** TENG based on SFA e-textile. **e** Vertical burning test of SFA e-textile using an alcohol lamp flame. **f** Output voltage change of SFA e-textile TENG at different temperatures. **d**–**f** Reproduced with permission from Ref. [74], Copyright 2022, American Chemical Society. **g** Preparation of carbonized aerogel. **h** Output electrical properties of FR-TENG at different combustion times. **i** Output voltage of FR-TENG at different temperatures. **g**–**i** Reproduced with permission from Ref. [72], Copyright 2019, Elsevier

Poly (p-phenylene benzobisoxazole) (PBO) is a highly stable refractory material with a decomposition temperature of up to 650 °C. A PBOA/PEO-TENG was fabricated using a PBO aerogel (PBOA) as the tribo-negative layer and poly (ethylene oxide) (PEO) as the tribo-positive layer material (Fig. 12a) [151]. The electrical output properties of the TENG remained stable when the temperature was increased from room temperature to 350 °C (Fig. 12b, c). Compared to traditional tribo-negative materials such as polytetrafluoroethylene (PTFE) and PDMS, PBOA has a much wider operating temperature range. Figure 12d shows an aerogel-fiber-based self-powered fire alarm e-textile (SFA e-textile) containing a TENG [74]. Aerogel fibers were produced by integrating Fe_3_O_4_ nanoparticles (Fe_3_O_4_NPs) and silver nanowires (AgNWs) into a calcium alginate hydrogel, followed by solvent replacement and freeze-drying. Compared with regular cotton fibers, the SFA e-textile demonstrates enhanced flame-retardant properties and thermal insulation (Fig. 12e). SFA e-textile has the potential to be utilized in firefighting clothing to monitor the surface temperature. When the temperature increased from 25 to 250 °C, the output voltage of SFA e-textile decreased but normal operation was maintained (Fig. 12f). Additionally, Fig. 12g shows the preparation of a carbon-based nanocomposite aerogel [72]. First, crystalline polymerization and sol–gel reactions of GO nanosheets, formaldehyde, resorcinol, and electrospun polyacrylonitrile (PAN) nanofibers were used to prepare a hydrogel. Subsequently, the hydrogel was subjected to supercritical drying and carbonization to produce a carbonized aerogel. The FR-TENG assembled with this aerogel exhibited excellent flame retardancy and high-temperature resistance (Fig. 12h, i). The FR-TENG maintained excellent output stability below 200 °C, as well as self-extinguishing functions.

#### Other Properties

**Fig. 13:**
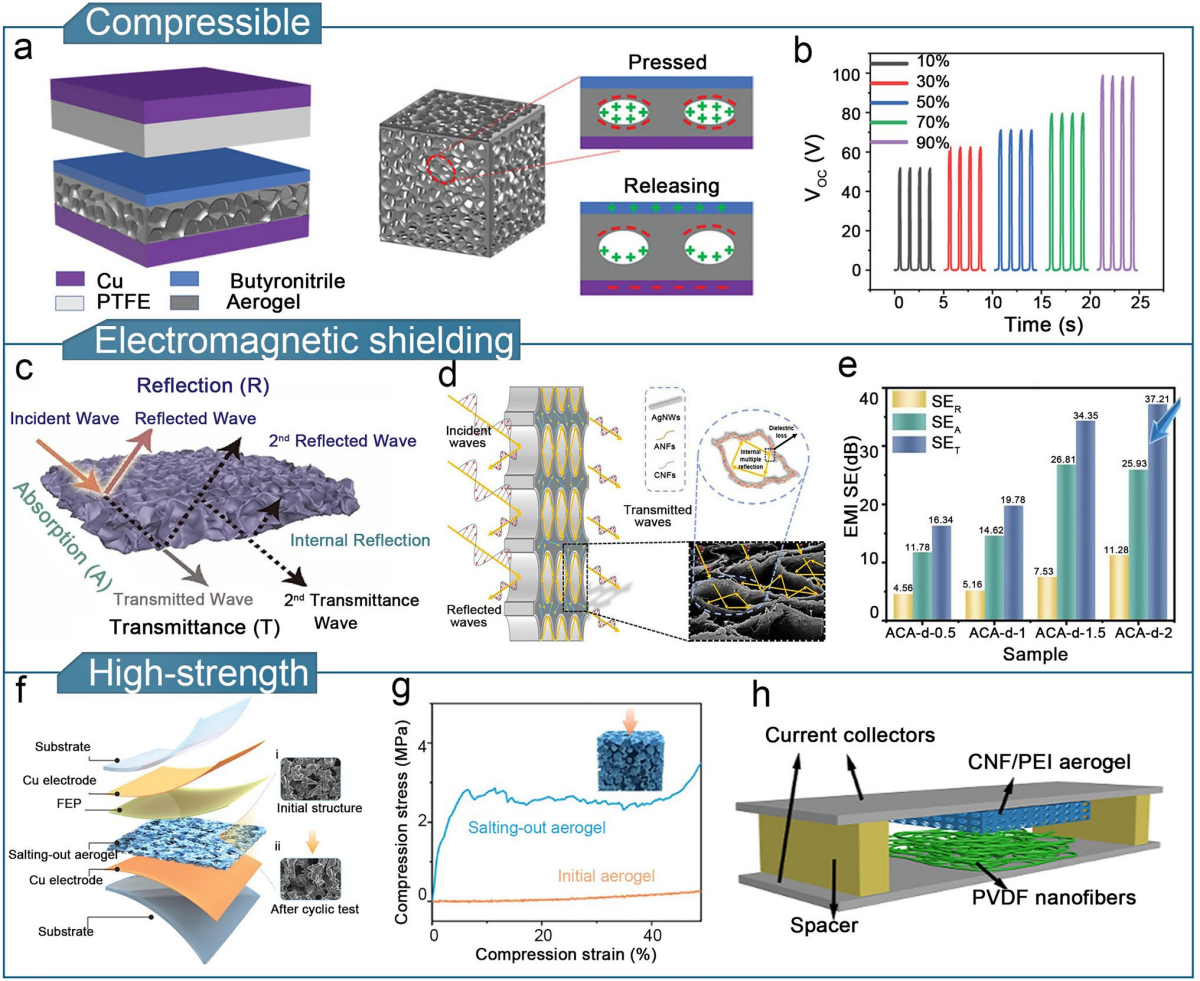
Optimization of other properties of aerogel-based TENGs. **a** Basic composition and working mechanism of gas solid TENGs. **b** Output voltage of a gas solid TENG at different compressive strains. **a**, **b** Reproduced with permission from Ref. [150], Copyright 2022, Wiley–VCH. **c** Electromagnetic shielding mechanism of MXene Ti_3_C_2_T_X_/CMC aerogels. Reproduced with permission from Ref. [166], Copyright 2022, Elsevier. **d** EMI shielding mechanism of ACA aerogel films. **e** Comparison of EMI shielding efficiency of ACA aerogel. SE_T_, SE_A_, and SE_M_ represent the total electrical shielding efficiency, shielding efficiency of microwave reflection, and shielding efficiency of microwave absorption, respectively. **d**, **e** Reproduced with permission [232]. Copyright 2023, Elsevier. **f** TENG structure prepared using a salting-out-treated cellulose aerogel. **g** Compressive stress strain curve of salting-out-treated cellulose aerogel. **f**, **g** Reproduced with permission from Ref. [86], Copyright 2023, Wiley–VCH. **h** Structure of CNF-PEI aerogel-based TENG. Reproduced with permission from Ref. [31], Copyright 2018, Elsevier

In addition to the aerogel-based TENGs previously discussed, aerogels with compressibility and elasticity are particularly suitable for fabricating flexible sensors because of their extensive deformation range and high contact area [42]. For example, GO/carboxy multi-walled carbon nanotube (GO/CMWCNT) hybrid aerogels were demonstrated [150]. Owing to the good elasticity and compressibility of the aerogel, the conductive inner surface was alternately loaded and unloaded with external pressure to achieve contact and separation. The assembled gas–solid TENG demonstrated stable performance over 20,000 loading/unloading cycles (Fig. 13a, b). The TENG assembled with this aerogel demonstrated the potential for use in human physiology and motion monitoring applications.

Electromagnetic interference (EMI) can have detrimental effects on the accuracy of sensing systems, especially in the case of TENG-based wearable sensors, where noise interference caused by electromagnetic waves reduces the reliability of sensor operation and can also affect human health. EMI can be mitigated by designing barriers made of conductive or magnetic materials. Porous aerogels with high electrical conductivity are resistant to microwave irradiation [166, 232]. Aerogel-based TENGs with EMI shielding capabilities can limit the signal interference-induced sensitivity damage to flexible sensors. An MXene Ti_3_C_2_T_X_/CMC aerogel prepared by using freeze-drying exhibited good EMI shielding performance (80.36 dB) owing to the electrical conductivity provided by the MXene Ti_3_C_2_T_X_ nanosheets [166]. The EMI shielding effect is achieved by the attenuation and dissipation of electromagnetic waves through multiple reflections and scattering between the pores and pore walls (Fig. 13c). The single-electrode TENG assembled with the MXene Ti_3_C_2_T_X_/CMC aerogel as the tribolayer was demonstrated for health monitoring and EMI shielding. Furthermore, ANFs/CNFs/AgNWs (ACA) aerogels prepared by directional freeze-drying and moderate compression can effectively control the pore structure and electrical conductivity of the aerogels [232]. As the AgNWs content increased, the EMI shielding performance of the ACA aerogel significantly increased up to a maximum of 34.34 dB (Fig. 13d, e). TENGs containing aerogel-film electrodes have the potential for use in health monitoring, EMI shielding, and sensing applications.

It should be noted that although aerogels are compressible, their structures are also prone to collapse, which limits their use in applications where they are exposed to significant deformation. The formation of hierarchical pore structures is an effective and commonly used strategy to meet the demands for high-strength aerogels [245, 246]. Nanocellulosic triboelectric aerogels were prepared by multiscale structuring via the Hofmeister effect [86]. Figure 13f shows the basic structure of a self-powered sensor assembled using this aerogel as a tribolayer. Based on the synergy between the Hofmeister effect and salting-out treatment, the compressive strength of the aerogel was significantly higher than that of the control group (Fig. 13g). In addition, amino-acid modification effectively improves the mechanical strength of cellulose aerogels. TENGs comprising a CNF-PEI (polyethylenimine) aerogel as the tribo-positive layer and polyvinylidene fluoride (PVDF) as the tribo-negative layer as shown in Fig. 13h [31]. The incorporation of 20% PEI effectively enhanced the mechanical properties of the CNF–PEI aerogel, resulting in a 60.8% increase in the tensile strength and a 237.4% increase in the compression modulus compared to the pure CNF aerogel. In addition, compared to the pure CNF aerogel, the aerogel-based TENG with 10% PEI showed a 14.4-fold increase in power density.

Overall, the optimization of gel-based TENGs has achieved the development requirements of flexible sensors in terms of device assembly and output performance. Table 2 summarizes the material optimization and the output characteristics of three types of gel-based TENGs. In gel-based TENGs, the three-dimensional skeleton components of gel are mostly composed of polymers (such as PAA, PVA, and PAM), carbon-based materials, and biomass materials (cellulose, chitosan, etc.). Among them, PAA, PVA, and PAM are the most widely used in hydrogel- and organogel-based TENGs, mainly because these polymers have advantages such as good biocompatibility, high transparency, adjustable elasticity, and simple synthesis processes [247]. In addition, the high water solubilities of PAA, PVA, and PAM facilitate the performance enhancement of hydrogel-based TENGs. For example, adding conductive substances [248], polysaccharides [197], or protein polymers to PAM [249] and strenghening network structures based on hydrogen bonds or intermolecular forces significantly improves the conductivity, tensile performance, and durability of hydrogels, enhancing their application advantages as electrodes in flexible TENG sensors.

**Table 2:** Optimized material properties and output performance of gel-based TENGs

Types	Property	Basic Materials	Key Parameters	*V*_oc_ (V)		*I*_sc_ (μA)	Refs
Hydrogel	Conductivity	PAA/sodium alginate	σ: 0.34 S m^−1^	30	0.5		[250]
		CMC/MOF/PVA	σ: 2.42 S m^−1^	269	17.9		[142]
		PAM/Lignin	σ: 1.22 mS cm^−1^	265	2.7		[251]
	Mechanical performance	PAM/HEA/ZIF-8	Strain: 570%; Stress: 88 kPa	232	–		[99]
		PVA/PAM/PAA	Tensile stress: 2.1 MPa; Toughness: 6.5 MJ m^−3^	238	1.2		[87]
		Starch/HEMA	Compressive strength: 6.83 MPa	87	0.59		[143]
		PAM/Clay	Healing within 1s (− 30 to 80 °C)	157	16		[192]
		PAA/GA	Healing efficiency: 94.7%	123	5.1		[144]
		PVA/PAM/CNC	Healing efficiency: 97.4%	0.3	–		[98]
		PVA/Sodium tetraborate	Full healing in 30 min	20	–		[194]
	Self-cleaning	HDFS	Average transmittance: 94.4%	277	–		[139]
	Frost-resistance	PAM/HEC	Frost-resistance: − 69 °C	285	15.5		[197]
		PAM/PVA	Frost-resistance: − 60 °C	200	13.1		[198]
Organogel	Toughness	CNF/PVA/SA	Toughness: 24.5 kJ m^−2^	145	8.7		[80]
		PIL-BF_4_/BTCA	Tensile stress: 2.28 MPa	–	0.2		[79]
		ACMO/PUA/EMIM/LiTFSI	Tensile strength: 2.29 MPa Breaking strain: 1062%	101	1.32		[81]
		PVDF/EMIm TFSI	Toughness: 37.5 kJ m^−2^	10	0.05		[83]
	Temperature tolerance	PAM/Montmorillonite/CNT	Temp: − 60 to 60 °C	86.4	1.1		[75]
		PEGDA/PAM/PVP	Temp: − 30 to 60 °C	160	7		[153]
		PAA/DMAPS/ [EMIM][OAc]	Temp: − 30 to 40 °C	48	4		[221]
		PAA/[EMI][DCA]/ DMAPS	Temp: − 20 to 100 °C	120	15		[222]
	Humidity resistance	PMEA/PIBA/[C_2_mim][NTf2]	Weight retention: 100% (90%RH, 240 h)	4	0.4		[157]
	Leak-proof	PC/LiTFSI	Water retention: 97% (after 30 d)	44	–		[77]
	Interfacial adhesion	PC/ PACMO	Interface peeling strength: 60 N m^−1^	87	–		[78]
Aerogel	Dielectric enhancement	CNF/CTS	Regulating porosity: 92%	60.6	7.7		[84]
		PUA	Regulating porosity: 33%	105.6	20.3		[73]
		PEI	Regulating porosity: 50%	40	5		[167]
	Thermal insulation	PBO/PEO	Temp: 350 °C	40	–		[151]
		CA/AgNWs	Temp: 250 °C	3.5	–		[74]
		PAN/rGO	Temp: 200 °C	80	–		[72]
	Compressibility	CNT/GO	Stable performance after 20,000 loading/unloading cycles	98.4	2.89		[150]
	Electromagnetic shielding	CMC/MXene Ti_3_C_2_T_x_	SE_T_: 80.36 dB	54.37	1.22		[166]
		CNF/ANF/AgNWs	SE_T_: 34.34 dB	100	1.52		[232]
	Srength	PVA/CNF	Young’s modulus: 142.9 MPa	–	6		[86]
		CNF/PEI	Tensile stress ~ 1.4 MPa	106.2	9.2		[31]
		CNF/PVA/CNT	Compression stress: 48.5 N	160	–		[152]
		CNF/PANI	Tensile stress: 104 MPa	130	–		[252]

CA, Calcium Alginate; [EMIM][OAC], 1-Ethyl-3-Methylimidazolium Acetate; EMIM/TFSI, 1-Ethyl-3-Methylimidazolium Bis(Trifluoromethyl Sulfonyl) Imide; MOF, Metal–Organic Framework; PACMO, Poly (4-Acryloyl Morpholine); PAN, Polyacrylonitrile; PANI, Polyaniline; PC, Propylene Carbonate; PEGDA, Polyethylene Glycol Diacrylate; PUA, Polyurethane Acrylate; PVP, Polyvinylpyrrolidone; σ, Electrical Conductivity; Temp, Temperature

Similarly, similar polymers are also widely used in organogel-based TENGs, and their functions and mechanisms are similar to those of hydrogels, with the main difference being that the solvent system changes from an aqueous phase to an organic solvent or ionic liquid. This transition in the solvent system can effectively solve the problem of decreased mechanical and conductive properties caused by dehydration in traditional hydrogels, making TENGs better suited for flexible sensing requirements in extreme environments. Carbon-based materials are mainly used as functional additives doped into the gel matrix to enhance the conductive, mechanical, and temperature resistance properties of gel-based TENGs, with similar mechanisms and effects among the three types of gel materials. Cellulose (CMC, CNF) can be obtained as environmentally friendly hydrogels through dissolution or cross-linking, further transformed into organic gels through solvent replacement, or prepared as aerogels through drying. Cellulose mainly serves as a skeletal support in gels, but since it is also a good triboelectric positive material itself, cellulose-based aerogels can be used as friction layers in TENGs [84, 165]. The discussion above highlights the differences in the performance and applications of the different types of gels made from the same raw materials. To promote the application and development of gel-based TENGs for flexible sensing, it is important to develop strategies to design and manufacture traditional gel materials with appropriate modifications to meet the various application requirements.

## Gel-Based TENGs for Flexible Sensing Applications

A high-performance gel-based TENG should offer strength, conductivity, environmental resistance, and thermal insulation, to enhance the sensitivity, detection range, and device durability of flexible triboelectric sensors. The enhanced performance significantly broadens the potential applications of gel-based TENGs. Gel-based triboelectric sensors are lightweight and compact, and can detect physical stimuli such as limb movement, touch forces, temperature, and humidity as well as chemical compositions such as sweat and gas [253]. Gel-based TENGs have diverse applications in fields such as human motion sensing, tactile sensing, health monitoring, environmental monitoring, human–machine interactions, wound dressings, implantable sensors, and intelligent traffic monitoring.

### Human-Motion Sensing

With the widespread application of flexible TENG in the new generations of electronic products, various flexible stretchable devices have been developed, such as sports watches and smart bracelets. These devices can sense and monitor the motion status of the users, helping individuals keep track of their physical activity and achieve personal health goals. Motion monitoring sensors based on gel-based TENGs can convert motion signals into electrical signals, thereby realizing the monitoring of human motion, such as limb, joint, and muscle movements.

**Fig. 14:**
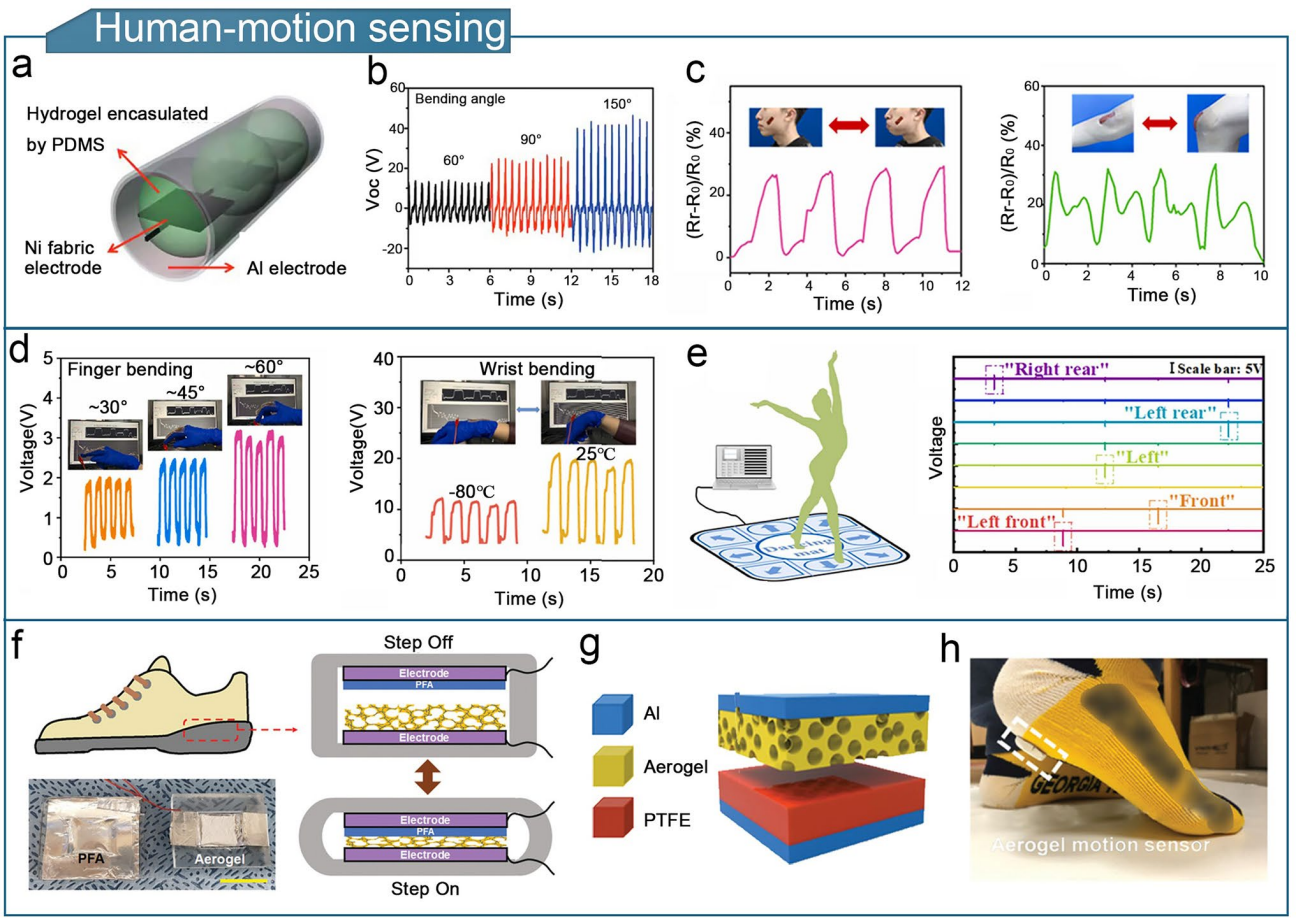
Gel-based TENGs for human motion sensing. **a** Structure of tubular PVA hydrogel-based TENG. **b** Voltage signal plot of PVA hydrogel-based TENG for monitoring bending. **a, b** Reproduced with permission from Ref. [65], Copyright 2017, Wiley–VCH. **c** Response signals of PAM/PAA hydrogel-based TENG monitored for cheek and cheek joint flexion. Reproduced with permission from Ref. [87], Copyright 2022, Elsevier. **d** Eutectogel-based TENG for finger flexion monitoring and wrist flexion monitoring at different temperatures. Reproduced with permission from Ref. [254], Copyright 2022, Elsevier. **e** Schematic diagram of a self-powered dance mat and its voltage signal for monitoring dance steps. Reproduced with permission from Ref. [88], Copyright 2023, Elsevier. **f** Schematic of PSI aerogel-based TENG mounted to an insole. Reproduced with permission from Ref. [255], Copyright 2022, Wiley–VCH. **g** Structure of cellulose aerogel-based TENG. **h** Schematic of attaching cellulose aerogel-based TENG to a sock. **g**, **h** Reproduced with permission from Ref. [240], Copyright 2020, Wiley–VCH

Owing to the unique properties of the human skin, it is crucial to select materials for motion sensors that exhibit excellent biocompatibility and degradability. A PVA hydrogel was prepared by freeze thawing, encapsulated in a hemispherical electrode with PDMS and nickel fabric, and assembled into a single-electrode TENG with aluminum electrodes (Fig. 14a) [65]. Interestingly, the PVA hydrogel was used as a flexible substrate rather than as an electrode or tribolayer. The PVA hydrogel-based TENG was utilized as a self-powered sensor to track quantitative data regarding the human body, including movement of the arms, knees, and throat (Fig. 14b). Given that PVA is an environmentally friendly material and does not require the addition of other chemicals, the PVA hydrogel-based TENG demonstrated good environmental performance, with fully recycled and remanufactured motion-sensor devices retaining up to 92% of the output of the original device. Furthermore, the interfacial adhesion and fatigue resistance of gel materials can significantly improve the sensitivity and applicability of motion monitoring. A conductive DNH was constructed using PVA and PAA–PAM as the first and second network, respectively. By leveraging the interactions between hydrogen bonds, free hydroxyl groups, and carboxyl groups, the mechanical and adhesion properties of the DNH were effectively enhanced [87]. The TENG using this hydrogel as the electrode, demonstrated high sensitivity and electrical output when monitoring the movement of the fingers, knees, cheeks, and elbows (Fig. 14c). In addition, a single-electrode TENG utilizing a eutectogel electrode was proposed for monitoring motion in subzero environments [254]. The eutectogel was composed of sulfonated lignin with Fe^3+^ and ammonium persulfate and fabricated by the double autocatalytic initiation of gelation, followed by the replacement of deep eutectic solvents (DESs). The eutectogel-based TENG demonstrated outstanding freeze resistance and maintained excellent electrical properties at temperatures as low as − 80 °C. This device was used to monitor limb movements in extremely cold weather, such as finger flexion and arm bending (Fig. 14d).

It is worth mentioning that aerogels are the preferred materials for the preparation of foot motion sensors owing to the performance advantages of their high porosity, light weight, compressibility, and resilience. For instance, a cellulose/CNT aerogel-based TENG (CCA-TENG) was used as both an electrode layer and a tribolayer to create a self-powered dance mat that translate a dancer’s movements into a voltage signal [88]. This enables the evaluation of the dancer’s strength of movement and foot placement, which can be used as a grading standard for formal contests or dance practices (Fig. 14e). Figure 14f shows a TENG based on a poly (succinimide) (PSI)-alginate aerogel [255]. The incorporation of PSI effectively improved the triboelectric properties and mechanical durability of the aerogel-based TENG. The TENG was integrated into an insole to assess the human locomotor gait by analyzing the force distribution between the left and right feet. Furthermore, the heels of socks containing the cellulose aerogel-based TENG produced a significant electrical output upon contact with the ground. (Fig. 14g, h) [240]. Owing to its light weight (5 mm thick), excellent mechanical flexibility, and high porosity, the cellulose aerogel-based TENG minimally interferes with the wearer’s gait and allows breathability.

### Tactile Sensing

With the emergence of smart industries, the popularity of electronic products with smart touchscreens has driven research on haptic interfaces. Touch sensing and recognition are crucial for the development and optimization of wearable electronic devices, electronic skin, and intelligent robots. Tactile sensing is essential for ensuring human safety through motion control, skill training, and the ability to recognize unknown signals. Gel materials have excellent flexibility and self-repair functionality, and are the preferred materials for the development of TENGs for tactile sensing. Gel-based TENGs in tactile sensors mainly respond to pressure to achieve energy conversion via the contact–separation mode [256].

**Fig. 15:**
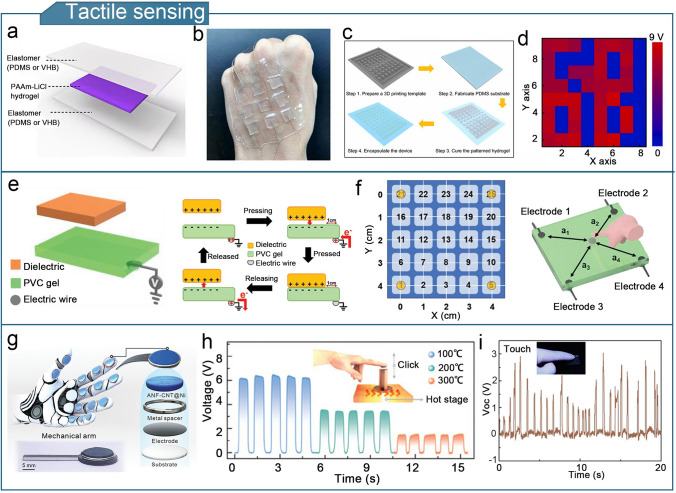
Gel-based TENGs for tactile sensing. **a** Structure of PAM-LiCl hydrogel-based TENG. **b** Schematic of PAM-LiCl hydrogel-based TENG for tactile sensors. **a**, **b** Reproduced with permission from Ref. [89], Copyright 2017, American Association for the Advancement of Science. **c** Fabrication process of an 8 × 8 tactile sensor array. **d** Electrical response signal when the word “GOOD” is written on the haptic sensing array. **c**, **d** Reproduced with permission from Ref. [256], Copyright 2022, Elsevier. **e** Structure and working mechanism of a single-layer TENG. **f** Schematic of PVC gel tactile sensor. **e**, **f** Reproduced with permission from Ref. [257], Copyright 2022, Wiley–VCH. **g** Schematic diagram of a high-temperature tactile sensor. **h** Output voltages are generated by a high-temperature tactile sensor when touching an object at different temperatures. **g**, **h** Reproduced with permission from Ref. [90], Copyright 2023, Wiley–VCH. **i** Electrical signals generated by TENG tactile sensors in response to finger touches. Reproduced with permission from Ref. [258], Copyright 2019, Elsevier

Initially, gel-based TENGs were developed as electronic skins for tactile sensing applications. A single-electrode hydrogel-based TENG was fabricated by encapsulating a PAM–LiCl hydrogel inside a PDMS elastomer and connecting it with wires (Fig. 15a) [89]. The PAM hydrogel exhibited superior stretchability (uniaxial strain up to 1160%) and transparency (96.2%), making it suitable for use in electronic skin to conformally fit the back of the hand (Fig. 15b). The PAM–LiCl hydrogel-based TENG was capable of sensing touch and pressures as low as 1.3 kPa. Additionally, a PAA–PAM ionic hydrogel doped with choline chloride and NaNO_3_ ions using interpenetrating polymer networks and ion doping was proposed [256]. This hydrogel exhibited high transparency (85%), electrical conductivity (1.243 S m^−1^), and stretchability (850%). The TENG assembled with the PAA/PAM ion-conducting hydrogel as an electrode was integrated with the skin to create an 8 × 8 tactile sensor array. Users were able to obtain the corresponding electrical signals by touching a position in the array and writing the word “GOOD” (Fig. 15c, d).

Typically, hydrogels and organogels require elastomer encapsulation to fabricate single-electrode TENGs. Therefore, it is important to consider the interfacial bonding between the gel and elastomer, which can require complex preparation processes. Simplifying the structure of the device improves the functionality of tactile sensors. Figure 15e shows the structure and working mechanism of a single-layer gel-based TENG composed of a polyvinyl chloride (PVC) gel modified by a plasticizer treatment which acts as both the electrode and tribolayer, and is grounded by wires [257]. The single-layer PVC-TENG is a simple and easy-to-fabricate structure with significantly enhanced material properties. The adipate plasticizer modification increased the transmittance of the PVC gel up to 91%, increased the dielectric constant by 90–300 times compared to that of the pure PVC gel, and improved the electrical conductivity. These advantages enabled the PVC-gel-based TENG to be integrated into tactile sensors to realize the tactile sensing of position and pressure (Fig. 15f).

In a recent study, a tunable anisotropically structured ANF aerogel was fabricated using a magnetically oriented self-assembly strategy (Fig. 15g) [90]. The aerogel exhibited excellent thermal stability, and a thermally stabilized anisotropic aramid triboelectric gel was assembled into a wearable self-powered sensor device to enable tactile sensing in a high-temperature environment at 300 °C (Fig. 15h). To address the problem of hydrogel dehydration, TENG tactile sensors were developed using an ionogel as the electrode and patterned PDMS as the tribolayer [258]. The high conductivity of the ionogel and excellent mechanical properties of PDMS provided the sensor with good stretchability (121%) and transparency (83%). The tactile sensor could detect finger touches, bending, blowing, and pulses (Fig. 15i). Furthermore, the sensor detected impact forces in the range of 0.1–1 N, with a maximum sensitivity of 1.46 V  N^−1^.

### Health Monitoring

Health monitoring is important for disease prevention, health management, personalized treatment, and health awareness. With the effective combination of wireless networks and sensing technologies, flexible triboelectric sensors provide effective tools for monitoring, assessing, managing, and improving health. Flexible triboelectric sensors integrated with gel material can sense and monitor internal physiological signals such as respiration, perspiration, blood pressure, and heart rate, which helps monitor the health status of the human body in real time [259].

**Fig. 16:**
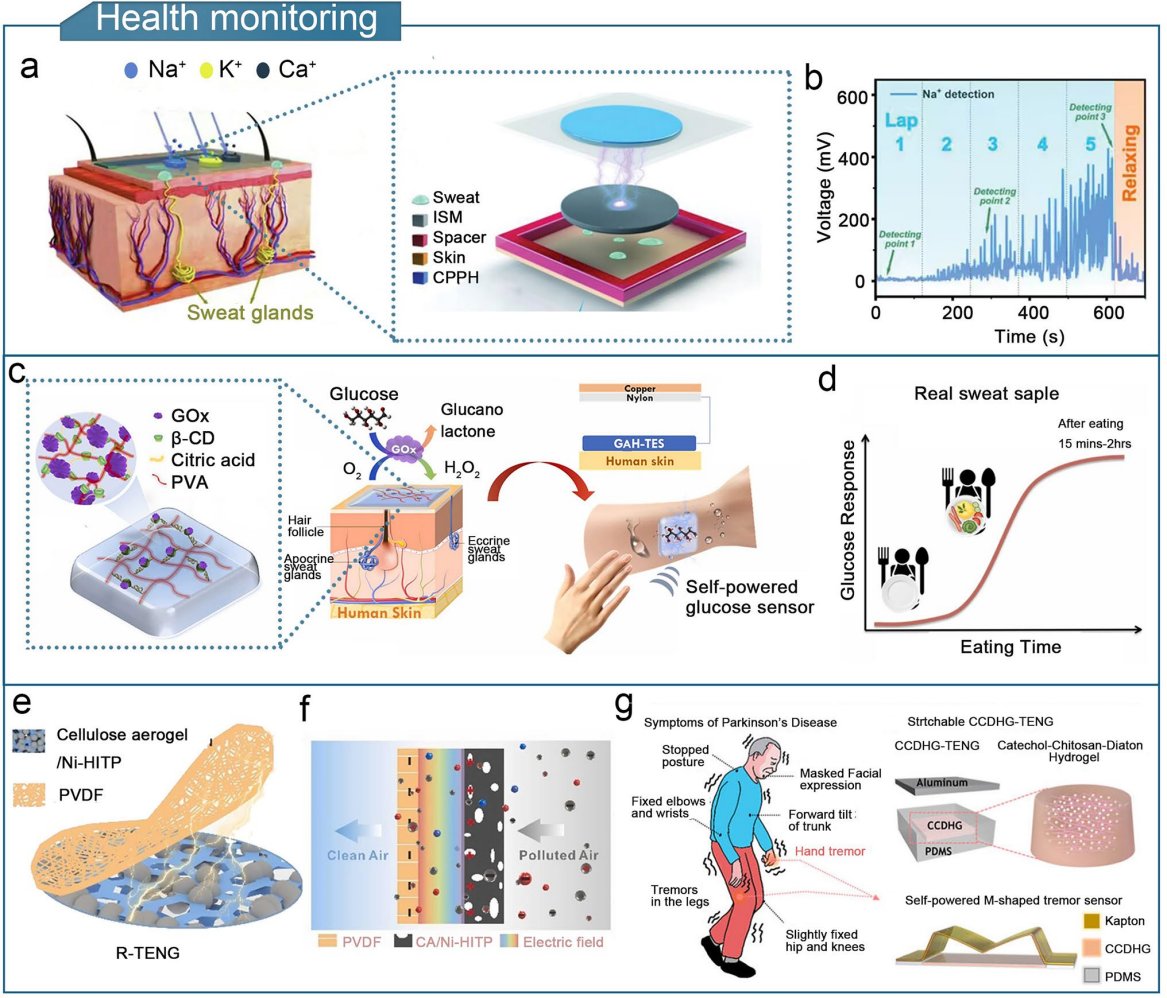
Gel-based TENGs for health monitoring. **a** CPPH as an electrode used as a sensor for monitoring Na^+^, K^+^, and Ca^2+^ elements in sweat. **b** Output voltage profile of CPPH for monitoring Na^+^ content in sweat. **a**, **b** Reproduced with permission from Ref. [91], Copyright 2022, Wiley–VCH. **c** GAH-TES for monitoring glucose levels in sweat. **d** GAH-TES to measure the amount of glucose in real sweat samples taken both before and after meals. **c**, **d** Reproduced with permission from Ref. [147], Copyright 2023, Elsevier. **e** Structure of R-TENG. **f** Filtration mechanism of self-powered air filters. **e**, **f** Reproduced with permission from Ref. [92], Copyright 2022, Elsevier. **g** CCDHG-TENG for Parkinson’s disease diagnosis. Reproduced with permission from Ref. [262], Copyright 2021, Elsevier

Sweat contains biomarkers such as electrolytes, metabolites, and trace elements [260], making it a valuable analyte for health monitoring. A triboelectric sweat sensor with a cellulose-based conductive hydrogel as the electrode was presented (Fig. 16a) [91]. The CPPH hydrogel formed by dynamic cross-linking between polyaniline (PANI) polymerized in situ with 2,2,6,6-tetramethylpiperidine-1-oxyl radical TEMPO-oxidized CNFs (TOCNF) and PVA/borax (PVAB). The sweat sensor was able to measure trace elements such as Na^+^, K^+^, and Ca^2+^ in sweat (Fig. 16b) and showed high sensitivity (K^+^ sensitivity down to 0.082 mmol^−1^). Monitoring glucose levels in humans is essential for managing diabetes, preventing complications, and assessing dietary health status. Monitoring of glucose levels provides an opportunity to understand and adjust glucose levels in a timely manner to maintain a healthy blood glucose range [261]. A self-healing glucose-adaptive hydrogel based triboelectric biosensor (GAH-TES) inspired by the enzymatic reaction of glucose was developed [147]. The GAH-TES consisted of a PVA matrix doped with β-cyclodextrin (β-CD)-encapsulated glucose oxidase. GAH-TES was highly selective and sensitive for measuring the glucose concentration in human sweat before and after a meal (Fig. 16c, d), and is suitable for the health monitoring of diabetic patients.

Furthermore, monitoring respiration can provide information on the general health of the user. Figure 16e shows a respiration-driven TENG (R-TENG) comprising a cellulose aerogel/conducting metal–organic framework (Ni-HITP) and a PVDF film [92]. The R-TENG was integrated into a mask to create a self-powered mask filter. Based on the stacked CNF with a large number of three-dimensional micro-nano-scale pores, together with the open metal sites on the Ni-HITP and electrostatic interactions, the filter enabled real-time monitoring of the respiratory status and can was demonstrated for the efficient filtration of submicron particles (Fig. 16f). Additionally, evaluating the health status of the human body requires monitoring sweat and respiration and diagnosing conditions. A catechol-chitosan-diatom hydrogel (CCDHG) was combined with an M-shaped Kapton film to develop self-powered tremor sensor [262]. The M-shaped Kapton film can shorten the contact–separation time during wire contact, which improves the electrical output and sensitivity of the sensor. This CCDHG-TENG sensor measured low-frequency vibratory movements of patients to assess the condition of individuals with Parkinson’s disease (Fig. 16g).

### Environmental Monitoring

Flexible sensor technology is playing a crucial role in global environmental management systems. Gel materials are excellent flexible sensing materials because they respond to various specific stimuli. To date, gel-based TENGs have been used to detect temperature, humidity, water quality, and gases in the environment.

**Fig. 17:**
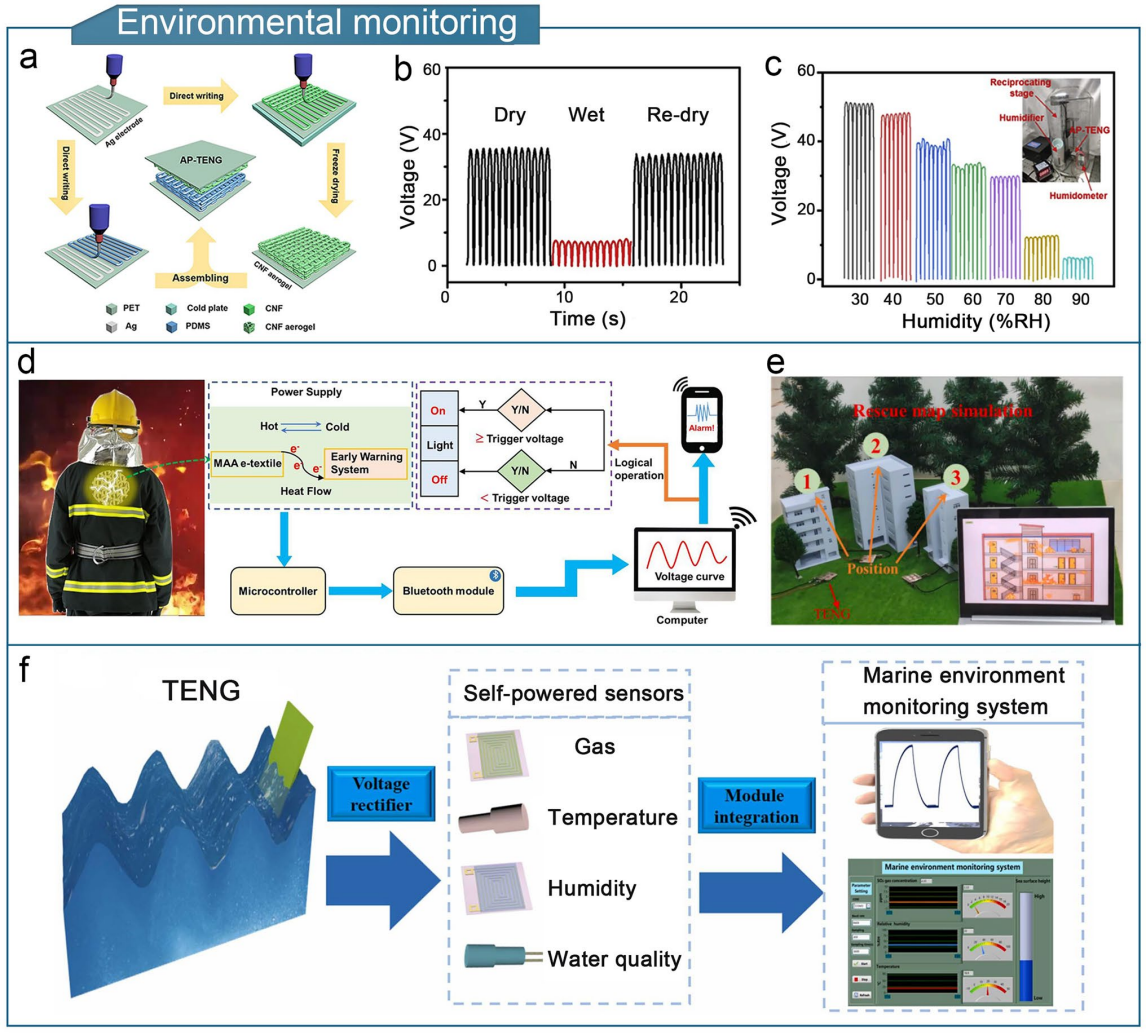
Gel-based TENGs for environmental monitoring. **a** Fabrication process of AP-TENG. **b** Voltage signals of AP-TENG humidity sensors in dry and humid environments. **c** Voltage response of AP-TENG humidity sensors in different humidity conditions. **a**–**c** Reproduced with permission from Ref. [165], Copyright 2019, Elsevier. **d** Operating mode of MAA aerogel-based TENG e-textile in fire warning system. **e** Schematic diagram of MAA aerogel-based TENG for the fire first aid simulation. **d**, **e** Reproduced with permission from Ref. [94], Copyright 2023, Elsevier. **f** Liquid–solid TENG for self-powered marine environment monitoring. Reproduced with permission from Ref. [93], Copyright 2022, Elsevier

The highly porous structure of the aerogels creates favorable conditions for water absorption. Therefore, aerogel-based TENGs are increasingly utilized for monitoring environmental humidity. An all-printed 3D hierarchically structured cellulose aerogel-based TENG (AP-TENG) is shown in Fig. 17a [165]. The 3D hierarchical micro/nanostructured cellulose aerogel provides a high contact area and surface roughness in the device, leading to enhanced electrical output. The AP-TENG was used in a self-powered humidity sensor with a response ratio up to 5:1, which sensed moisture-induced surface potential changes and monitored environmental humidity (Fig. 17b, c). Furthermore, aerogels are highly valued for their superior thermal insulation and flame-retardant properties, making them ideal for firefighting applications [121]. Figure 17d shows the working principle of TENG e-textiles based on MXene/AgNW/ANF (MAA) aerogel fibers for fire-warning systems [94]. ANFs combine the advantages of high-performance aramid and polymer nanofibers with excellent heat resistance, strength, and flame retardancy. Through the synergistic carbonization of MXene and ANFs, the MAA e-textiles exhibited significant flame retardancy and insulation. Figure 17e illustrates a fire first-aid simulation with three labeled locations to demonstrate the utility of the MAA aerogel-based TENG. The MAA textile sensed temperatures of 100–400 °C and was used to fabricate fire-resistant suits that provide a rapid alarm response within 1.6 s after being exposed to fire.

As human activities continue to expand, and industrialization accelerates, marine ecosystems are facing increasing threats and challenges. To maintain the ecological balance, it is crucial to enhance the detection of marine environmental conditions. To this end, a liquid–solid TENG was used in a self-powered sensing system for marine environmental monitoring (Fig. 17f) [93]. The liquid–solid TENG comprised an ethylene chlorotrifluoroethylene (ECTFE) film, a PVA-ethylene glycol hydrogel electrode, and a PVC substrate. Relying on the wave-driven effects on seawater levels, this gel-based TENG produces energy based on changes in the contact area of seawater on the ECTFE surface. The integrated processing of electrical signals from TENGs enables self-powered sensors to monitor environmental factors in real time, such as the SO_2_ concentration, temperature, humidity, and water quality, while facilitating the calibration of temperature and humidity to reduce errors in gas sensors. Triboelectric cellulose aerogels have also demonstrated advantages for applications in gas monitoring. By introducing heterogeneous interfacial engineering between TEMPO-oxidized CNF (TOCNF) and CNTs, a hierarchical porous structure and compressible triboelectric aerogel were fabricated [152]. The 3D porous microchannels of this aerogel provide many active sites that facilitate the adsorption and desorption of gases. The triboelectric aerogel exhibited sensitive monitoring of ammonia gas, with a detection range of 20–150 ppm, which is suitable for the food quality monitoring.

### Human–Machine Interactions

The joint development of wireless sensors and the Internet of Things requires improved systems for human–machine interactions, which are expected to realize autonomous perception and intelligent decision-making [263]. Flexible triboelectric sensors based on gel materials have made great progress in human–machine interaction, mainly in the development of human–machine interfaces, information protection and encryption, and intelligent robotic arms.

**Fig. 18:**
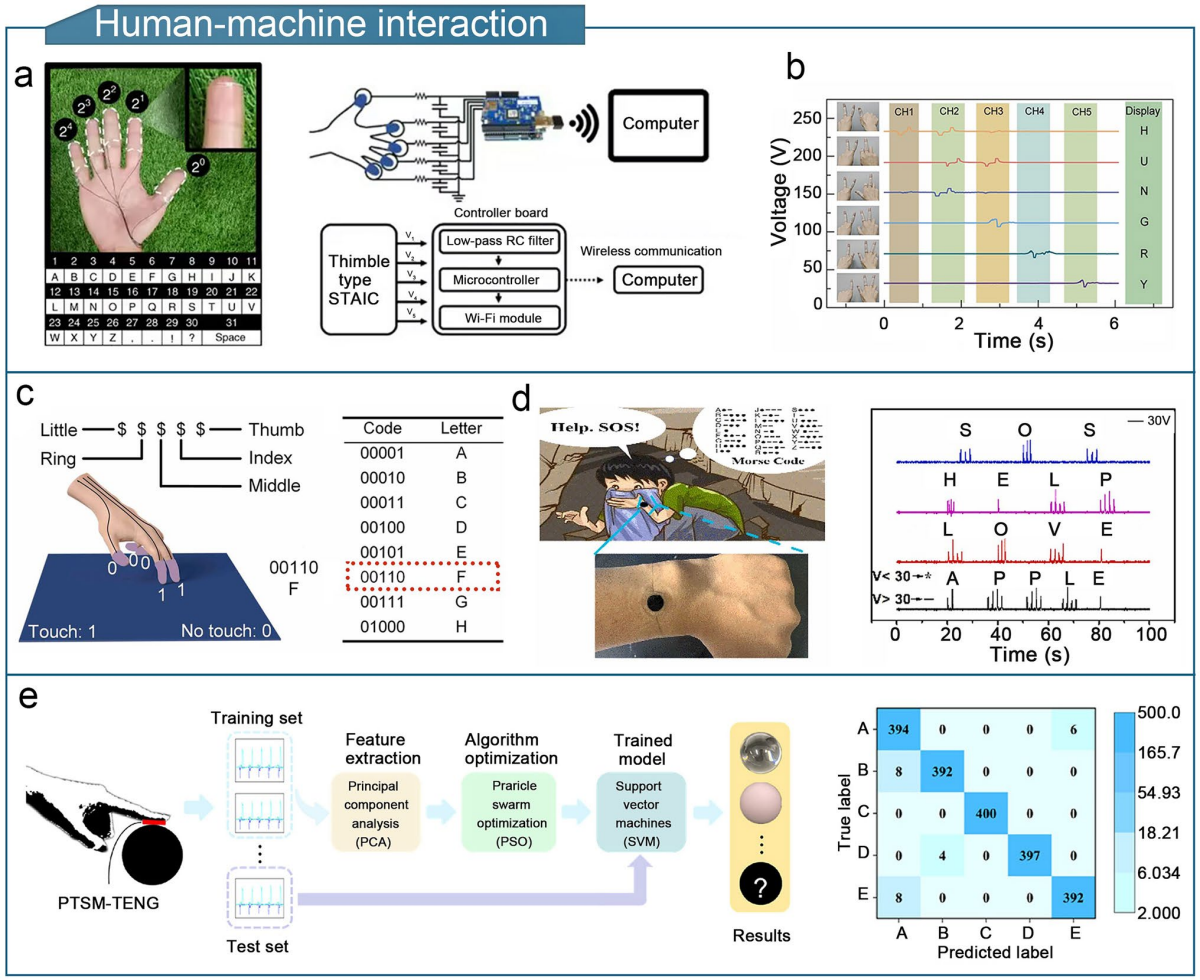
Gel-based TENGs for human–machine interactions. **a** TENG-based ion communicator for tactile sensing of gestures and applications in human–computer interaction. Reproduced with permission from Ref. [139], Copyright 2018, Springer Nature. **b** Output voltage of PVA/PA hydrogel-based TENG when expressing the term hunger with different combinations of gestures. Reproduced with permission from Ref. [95], Copyright 2023, Elsevier. **c** Antifreeze hydrogel-based TENG for binary conversion for gesture recognition. Reproduced with permission from Ref. [96], Copyright 2021, Elsevier. **d** PEDOT: PSS-PGA hydrogel-based TENG for Morse code encrypted communication and output voltage. Reproduced with permission from Ref. [264], Copyright 2022, Elsevier. **e** Schematic diagram of PTSM-TENG for object recognition and confusion recognition test curves. Reproduced with permission from Ref. [265], Copyright 2023, American Chemical Society

Gel-based TENGs are inevitably contaminated by the environment under continuous friction operation, which reduces the effectiveness of human–machine interactions. To address this, the surface of a PAM–LiCl hydrogel was coated with HDFS, encapsulated with PDMS, and connected to wires to obtain hydrogel-based TENGs [139]. The HDFS coating improved both the hydrophobicity of the gels and the sensitivity of the devices. The self-cleaning behavior of the hydrogel-based TENG ensured the output stability of the device. Hydrogel-based TENG sensors were attached to each finger and the combined signals were interpreted as an alphabet by encoding the five finger sensors for processing, thereby promoting real-time communication using a microcontroller (Fig. 18a). In addition, a flexible and stretchable PVA/PA (phytanic acid) hydrogel-based TENG was developed and placed on human joints [95]. The slight bending motions of the patient’s fingers were converted into electrical signals by the TENG, which were connected to a cloud interface to express the word “hunger”. Hydrogel-based triboelectric sensors can be used in emergency distress calls. In response to the limited interfacial interaction ability of hydrogels at subzero temperatures, a PAM–clay organohydrogel was prepared using an ethylene glycol–water binary solvent [96]. Ethylene glycol acted as an antifreeze to maintain stable properties at − 30 °C by forming molecular clusters with water and disrupting the hydrogen bonding network. A single-electrode TENG assembled using this hydrogel was attached to a finger and recognized tapping motions on the contact panel. The binary code was converted into letters and punctuation marks to display a signal on the monitor (Fig. 18c).

Information encryption is essential for safeguarding privacy and upholding rights and interests. Gel-based TENGs can be used to achieve information security by connecting sensors to human fingers to decode communications and recognize gestures. Zhang et al. integrated poly (3,4-ethylenedioxythiophene):poly(styrene sulfonate)(PEDOT:PSS) into γ-polyglutamic acid (PGA) hydrogels to produce electrically-conducting γ-PGA/PEDOT:PSS hydrogels [264]. The extensive formation of hydrogen bonds between PGA and PEDOT:PSS greatly enhanced the mechanical characteristics, adhesion, and self-healing capacity of this hydrogel. Figure 18d shows the Morse code communication achieved by finger tapping with the hydrogel directly attached to human skin. Similarly, based on multiple hydrogen-bonding interactions, PAM/TA/SA/MXene hydrogels were prepared using a one-pot method [265]. With the DNH structure formed using PAM/SA and tannic acid, coupled with the synergistic effect of MXene, the hydrogel exhibited enhanced mechanical properties and electrical conductivity. The PTSM-TENG was prepared using a PAM/TA/SA/MXene hydrogel as the electrode and integrated with a microcontroller as a wireless control system for a robotic hand. The robotic glove achieved object recognition and classification by gathering feature information from five spherical objects through touch. The confusion matrix test results indicated an accuracy of 98.7% (Fig. 18e).

### Other Applications

**Fig. 19:**
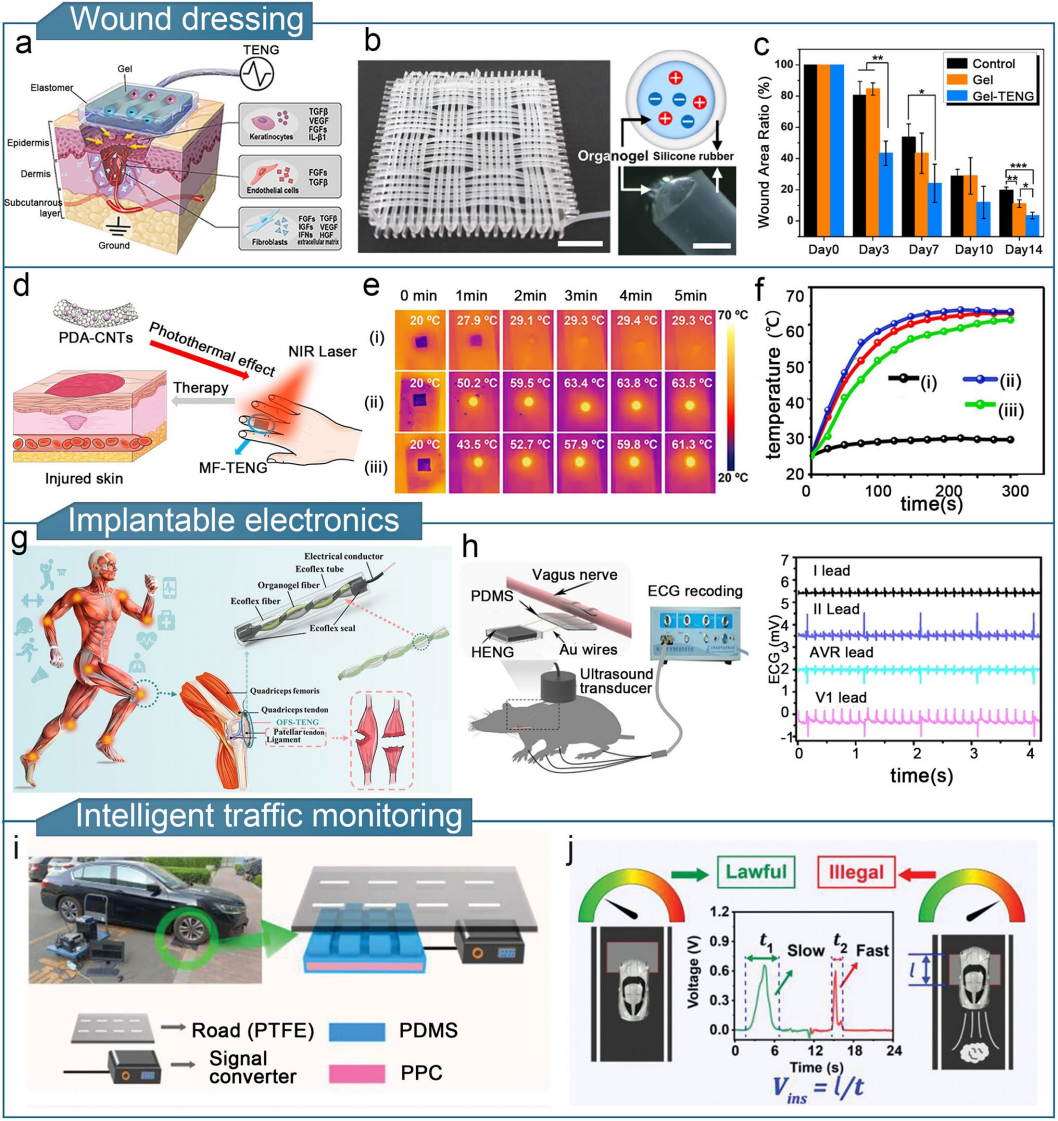
Other fields. **a** Organogel-based TENG for ionic patches. **b** Organogel-based TENG for ionic fabrics. **c** Remaining wound area after 3, 7, 10, and 14 days of treatment with ionogel-based TENG. **a**–**c** Reproduced with permission from Ref. [97], Copyright 2021, Elsevier. **d** Schematic of PDA-CNTs/PVA hydrogel-based TENG for photothermal wound treatment. **e** Photothermal images of TENG made with (i) pure PVA, (ii) PDA-CNTs/PVA hydrogel, and (iii) MF-TENG as electrodes under near-infrared (NIR) laser irradiation. **f** Photothermal contrast curves. **d**–**f** Reproduced with permission from Ref. [164], Copyright 2021, American Chemical Society. **g** Structure of OFS-TENG and its application to knee ligament monitoring. Reproduced with permission from Ref. [76], Copyright 2022, American Chemical Society. **h** Schematic diagram of HENG measurements of rat vagal electrocardiogram (ECG) and signal response plots. Reproduced with permission from Ref. [146], Copyright 2021, Elsevier. **i** Structure of the PPC-TENG sensor. **j** Changes in the output signal of the PPC-TENG to monitor the vehicle speed. **i**, **j** Reproduced with permission from Ref. [98], Copyright 2023, Wiley–VCH

Gel-based TENGs also have applications beyond the five fields mentioned above. Hydrogels and organogels, which are biocompatible and have a cell structure similar to that of human tissues, have shown significant potential in wound healing and implantable self-powered devices. An ionic TENG (iTENG) patch that served as both an electrode and a wound dressing was developed [97]. The patch is composed of an organogel encapsulated within an elastic membrane. The iTENG accelerated wound healing through electrical stimulation (Fig. 19a–c). Additionally, a hydrogel was fabricated by incorporating sodium borate and CNTs modified with polydopamine into PVA, which was used as a single electrode in a TENG [164]. When mechanically damaged, the TENG device repaired itself within 10 min at room temperature because of the repairable network of dynamic imine and borate ester bonds. This design has the potential for use in photothermal therapy to restore human joint motion under near-infrared laser irradiation (Fig. 19d–f).

Implantable flexible sensors are an important development for in-vivo medical applications. Unlike the common in-vitro flexible sensors, implantable flexible sensors are capable of in-vivo disease monitoring and provide more accurate and effective information for treatment [266]. Triboelectric sensors based on gel materials with similar structures to those of human tissues, flexibility, and biocompatibility, have demonstrated advantages for in-vivo disease treatment. For example, a TENG composed of organogel and silicone fibers (OFS) entangled in a double-helix structure was demonstrated (Fig. 19g) [76]. The OFS-TENG was implanted into the patellar ligament of a rabbit knee for real-time monitoring of knee ligament pull and muscle stress to assess muscle injury status and recovery training. Moreover, in-vivo and in-vitro tests of an organogel-based triboelectric sensor showed that it provided a proliferative microenvironment for cardiomyocytes with good biocompatibility. A high-performance hydrogel nanogenerator (HENG) was developed with polyacrylamide/graphene hydrogel electrodes [146]. The HENG is a novel liquid-based TENG that can be integrated into an implantable self-powered neurostimulator. The neurostimulator was implanted subcutaneously into rats and stimulated their vagus nerves to respond to ultrasound-driven stimulation (Fig. 19h), and showed the ability to inhibit the growth of proinflammatory cytokines, which could be used as an anti-inflammatory treatment for sepsis.

Furthermore, gel-based TENGs have demonstrated distinct advantages in multifunctional intelligent transportation. For instance, a PL-TENG was fabricated using a PAM–LiCl hydrogel as an electrode and PDMS as an elastomer tribolayer [267]. Attaching the PL-TENG to a driver’s face and neck enables the assessment of driver fatigue and distraction by monitoring motion signals such as eye closure, mouth closure, and neck rotation. Functionalized gel-based TENGs were developed for intelligent traffic monitoring of vehicle speed [98]. A DNH named PPC composed of PVA-PAM and tannic acid-modified cellulose nanocrystals was developed. A TENG was fabricated using a PDMS-encapsulated PPC hydrogel as the tribo-positive layer as well as an electrode, and PTFE as the tribo-negative layer (Fig. 19i). The developed sensor could monitor vehicle speed and weight while exhibiting long-term output stability (Fig. 19j).

## Summary and Outlook

The unique three-dimensional dynamic network structure enables gels to be tailored to meet the diverse requirements of flexible sensing devices in terms of material conductivity, flexibility, environmental adaptability, and biocompatibility. The emergence of gel-based triboelectric flexible sensors has promoted the development of self-powered wearable and flexible sensors. This review comprehensively summarized the recent research progress in gel-based TENGs for flexible sensing from the perspectives of principles, performance, and applications. The flexible triboelectric sensors comprising the three types of gel materials each have their own characteristics and advantages, can be tailored for various sensing applications and have demonstrated extraordinary research value in flexible TENG research.

Although gel-based TENGs have been extensively studied for flexible sensors, many challenges remain. Therefore, the optimization of the materials and triboelectric properties remains an effective way to consolidate the development of gel-based TENGs for flexible sensors. This section discusses the existing challenges of gel-based TENGs for flexible sensors in terms of materials, electrical output properties, and proposes feasible strategies for broadening their application potential based on existing research.

### Material Challenges of Gel-Based TENGs

Although the material performance of gel-based TENGs has shown significant progress, obtaining gel materials with excellent overall performance remains a major research focus. The incorporation of conductive polymers and carbon-based fillers can effectively increase the conductivity of gels used as electrode materials. However, this may lead to poor transparency, which does not meet the design requirements for electronic skin and implantable sensors [140]. In addition, the gel material must exhibit excellent mechanical properties, such as high flexibility, elasticity, and durability, to withstand various impact deformations encountered during sensor operation. High-strength gel-based TENGs based on energy-dissipation mechanisms often suffer from excessive aggregation of the conductive filler. This affects the ion-transport channels and reduces their conductivity [87]. Furthermore, challenges related to solute loss in hydrogels and inadequate anti-freeze and water retention capabilities have significantly affected the sensitivity and durability of flexible triboelectric sensors [197]. To meet the requirements of various challenging applications, the design of environmentally tolerant organogel-based TENGs requires improving their resistance to high humidity and solvent corrosion [224]. Furthermore, it is necessary to consider the balance between the flexibility, biodegradability, and environmental tolerance of a material. Aerogel-based TENGs have the advantages of high porosity, compressibility, and high specific area, leading to high triboelectric output and excellent deformation modulation. However, the aerogel structure is prone to collapse, and its strength must be increased to improve the mechanical durability and sensitivity of flexible triboelectric sensors. To reduce the environmental impact of flexible sensors, it is important to optimize the performance of gel-based TENGs based on biomass materials [268, 269]. Therefore, future research should focus on developing gel materials with outstanding overall performance to satisfy the application requirements of flexible triboelectric sensors in challenging environments.

### Optimization of the Output Performance of Gel-Based TENGs

Compared with TENGs assembled with conventional metallic materials, the output of gel-based TENGs still requires improvement. Previous studies have focused on enhancing the design to promote triboelectric charge generation and storage. Strategies for achieving this include increasing the surface charge density through the surface microstructure [165], nanofiller addition, and surface functionalization. Additionally, the optimization of the device structure by utilizing a two-electrode mode [89, 145, 270–272], monolayer TENGs [257, 270], integration with piezoelectric/piezoresistive sensors [238, 273–276], and power management are studied have been studied [277, 278]. Among these, the optimization of device structures has not yet been widely studied. Although the two-electrode mode effectively increases the charge-conversion efficiency compared to single-electrode gel-based TENGs, such devices is not conducive to the integration with human skin, thus limiting their application in flexible wearable electronic devices. However, little progress has been made in enhancing the output of gel-based TENGs by suppressing triboelectric charge decay. The introduction of electret materials [277], multilayer structures [279], or intermediates can significantly enhance the electrical performance and suppress triboelectric charge decay. The development of strategies to further inhibit the decay of triboelectric charges in gel-based TENGs should promote their use in high-performance flexible sensors.

### Further Development of Gel-Based TENGs for Flexible Sensing

The application of gel-based TENGs has led to significant advancements in wearable electronics and implantable sensors. To maximize the potential of each gel type, it is necessary to develop hydrogel-based TENGs with smart responses. For example, the development of hydrogel-based TENGs with intelligent response capabilities is a feasible approach to broadening the applications of flexible TENGs in actuators, biomedicine, and other fields. The development of organogels for TENGs is expected to promote their application in monitoring food safety and cosmetic quality. Additionally, TENGs play a pivotal role in the development of self-powered gas sensors for environmental monitoring [280]. Aerogels with selective adsorption capacity can significantly enhance the sensitivity and accuracy of gas sensors, making them the preferred materials for gas sensitization. Therefore, the future development of aerogel-based TENGs for gas-sensing applications is strongly encouraged. Multimodal sensing is a developing trend in flexible sensing. Gel-based TENG research should focused on improving their selectivity, mechanical durability, and sensitivity to function as multimodal sensors, thereby increasing their practical value in flexible sensing.

Furthermore, the commercialization of gel-based TENGs is still in its early stages, owing to challenges in mass production and cost control. It is necessary to consider not only the device durability, production cost, and core technology, but also the market demand, consumer psychology, and marketing strategies to determine whether gel-based TENG flexible sensors can be industrialized. The commercialization of gel-based TENGs for flexible sensing is expected to mature and gain popularity as technology continues to progress and the market demand grows.

In conclusion, all three gels have unique performance advantages and exhibit extraordinary research value in the study of TENGs for flexible sensing applications. This review aims to guide future research on gel-based TENGs to promote their wider and deeper development in flexible sensing and other fields.
